# Supplementary material for: The human disease-associated gene ZNFX1 controls inflammation through inhibition of the NLRP3 inflammasome
Source: EMBO J. 2024 Sep 27;43(22):9. doi: 10.1038/s44318-024-00236-9 (PMC11574294; doi:10.1038/s44318-024-00236-9)
Supplement: Supplementary file 1 — Appendix [file 44318_2024_236_MOESM1_ESM.pdf]

The human disease-associated gene *ZNFX1* controls inflammation through inhibition of the NLRP3 inflammasome

Jing Huang, Yao Wang#, Xin Jia, Changfeng Zhao, Meiqi Zhang, Mi Bao, Pan Fu, Cuiqin Cheng, Ruona Shi, Xiaofei Zhang, Jun Cui, Gang Wan# and Anlong Xu#

#Correspondence

Table of content

Appendix Figure S1. Confirmation of ZNFX1's role in suppressing the activation of NLRP3 inflammasome in independent cell lines……………………………………………3

Appendix Figure S2. ELISA to measure IL-1β and TNF-α in the supernatant…………..4

Appendix Figure S3. ZNFX1 inhibits dispersed vesicle formation and NLRP3 translocation related to Figure 4……………………………………………………………5

Appendix Figure S4. ZNFX1 harboring patient-derived mutations or helicase mutants failed to inhibit NLRP3 translocation……………………………………………………….6

Appendix Figure S5. MCC950 alleviates excess inflammation-induced symptoms in the EAE model……………………………………………………………………………………7

Appendix Table S1. Primers for qPCR analysis…………………………………………….8

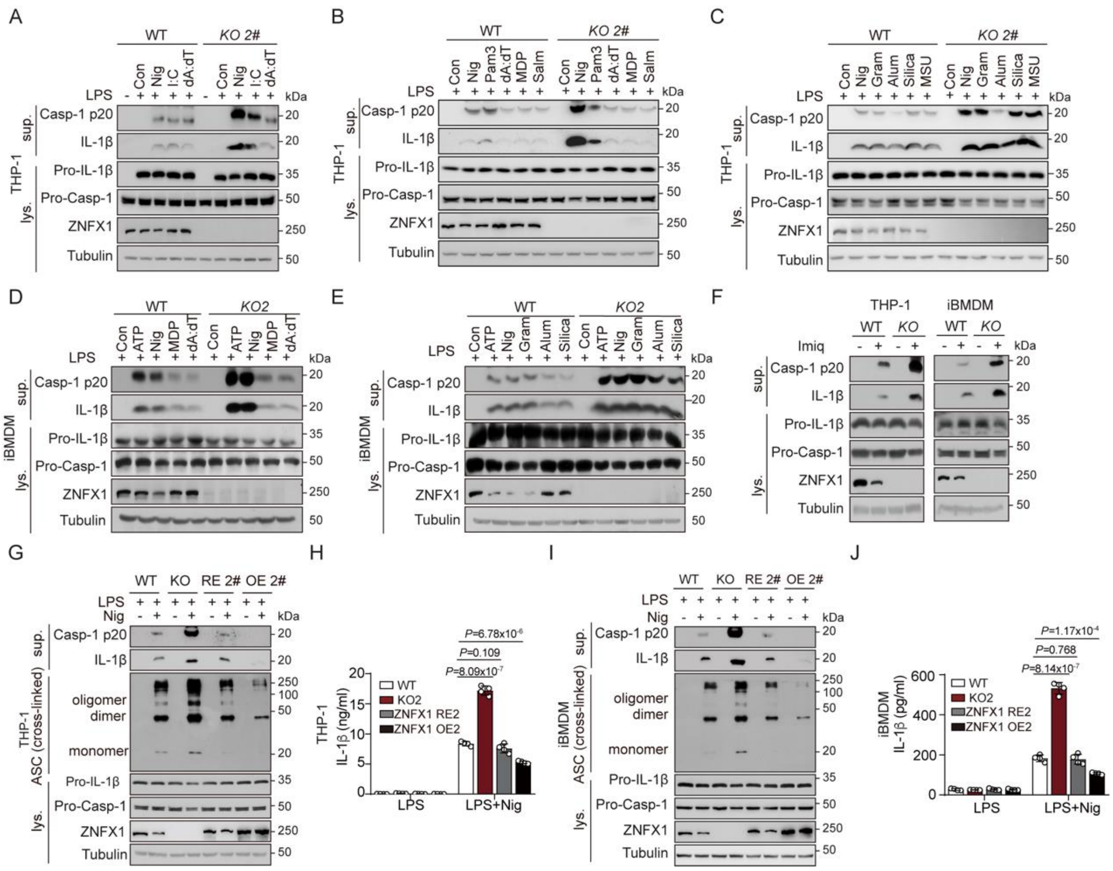

Appendix Figure S1. Confirmation of ZNFX1's role in suppressing the activation of NLRP3 inflammasome in independent cell lines. (**A-F**) An independent cell clone of WT and *ZNFX1* KO THP-1 derived macrophage or iBMDM was primed with LPS and then treated with indicated inflammasome agonists. Proteins in the medium supernatant and cell lysate were detected with immunoblot using indicated antibodies. (**G-J**) An independent cell clone of THP-1 derived macrophages (**G**) or iBMDM (**I**) RE and OE clone with indicated genotype were primed with LPS followed by control or nigericin treatment. ASC oligomerization and indicated proteins were measured by immunoblot (**G, I**). Secreted IL-1β in medium supernatant was measured with ELISA (**H, J**). n = 4 biological replicates. Error bars represent ± s.d.

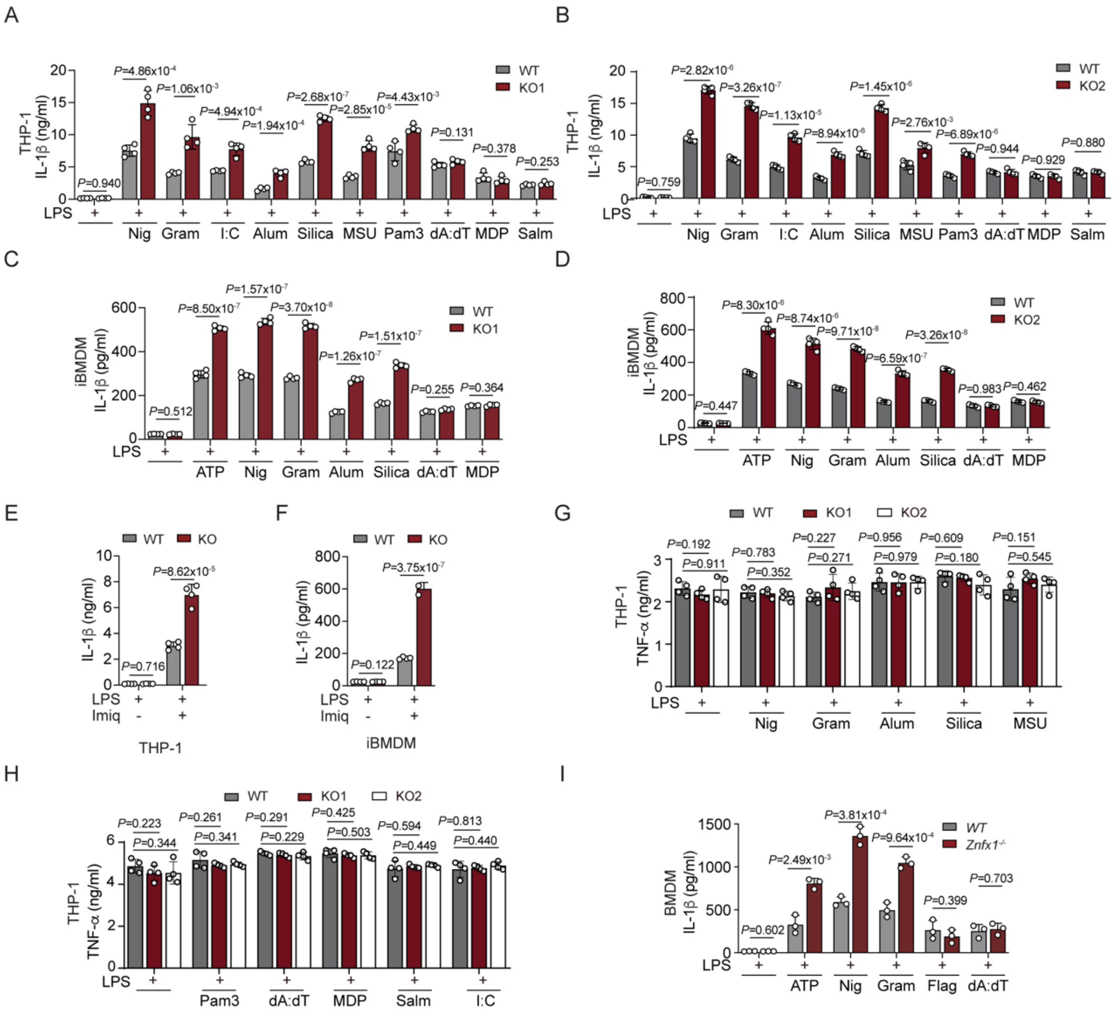

Appendix Figure S2. ELISA to measure IL-1β and TNF-α in the supernatant. (**A-I**) THP-1-derived macrophages, iBMDM, and primary BMDM cells were primed and treated with indicated inflammasome agonists. IL-1β and TNF-α in the supernatant were measured by ELISA.

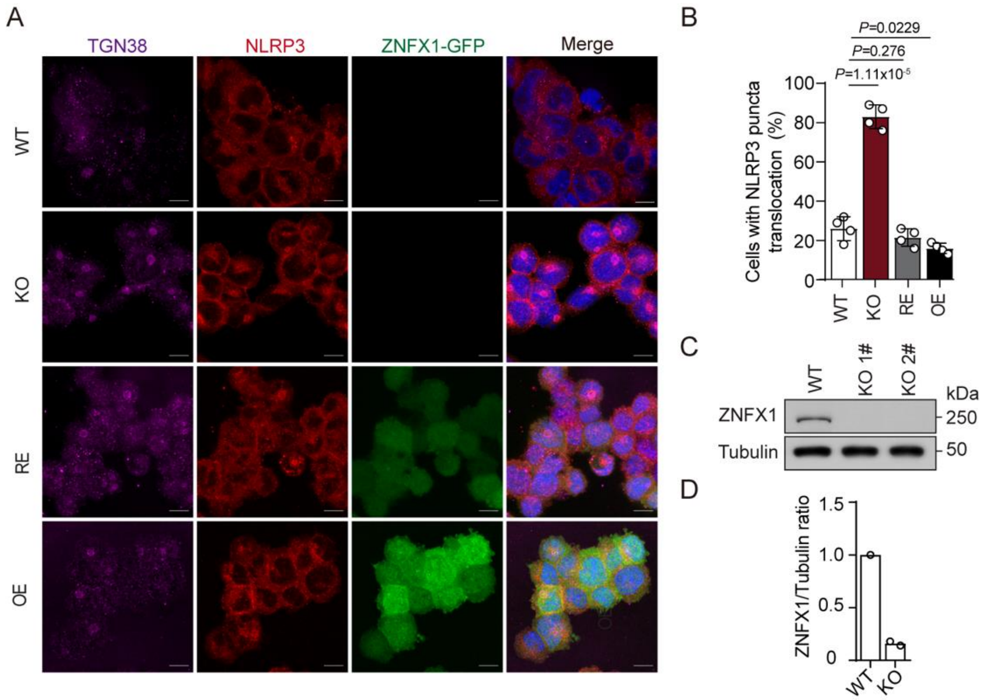

Appendix Figure S3. ZNFX1 inhibits dispersed vesicle formation and NLRP3 translocation related to Figure 4. (**A**) Immunofluorescence analysis of THP-1 derived macrophage with indicated genotypes using α-NLRP3 and α-TGN38 antibodies. (**B**) Quantification of NLRP3 translocation from 100 cells in (**A**). Data represent n = 4 biological replicates, mean ± s.d., two-sided Student's t-test. (**C-D**) Immunoblotting to detect ZNFX1 in WT and two independent colonies of *ZNFX1* KO HeLa cells (**C**). Quantification of the ZNFX1/Tubulin ratio from two biological replicates (**D**). For each biological replicate, band intensity was measured using ImageJ. The WT control was set to 1, and the ratio for *ZNFX1* KO cells was calculated by dividing their intensity by the corresponding WT control intensity.

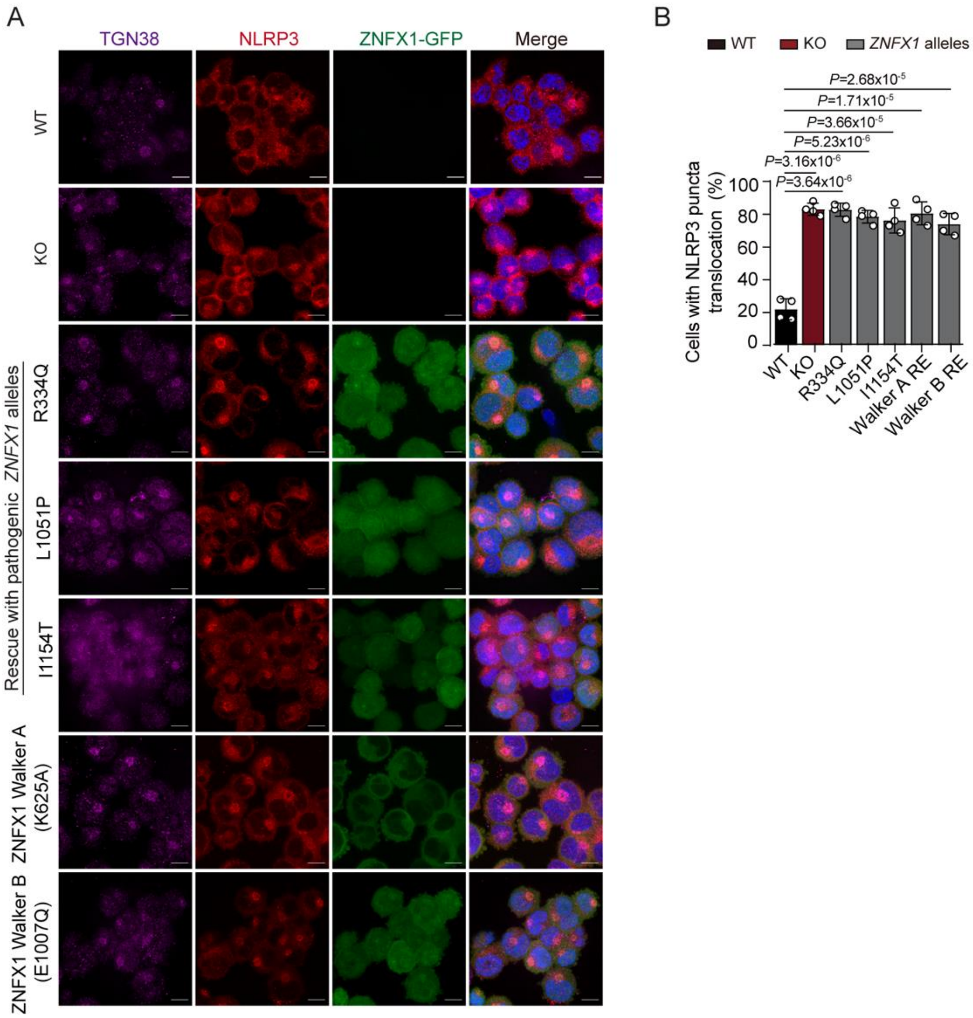

Appendix Figure S4. ZNFX1 harboring patient-derived mutations or helicase mutants failed to inhibit NLRP3 translocation. (**A**) WT, *ZNFX1* KO, or *ZNFX1* KO cells reconstituted with ZNFX1 containing ZNFX1 patient-derived mutations or helicase mutants in THP-1 derived macrophage were fixed and stained with anti-TGN38 and anti-NLRP3 antibodies. Scale bar, 10 μm. (**B**) Quantification of cells with NLRP3 translocated to TGN38+ vesicles from 100 cells in (**A**). Data represent n = 4 biological replicates, mean ± s.d., two-sided Student's t-test.

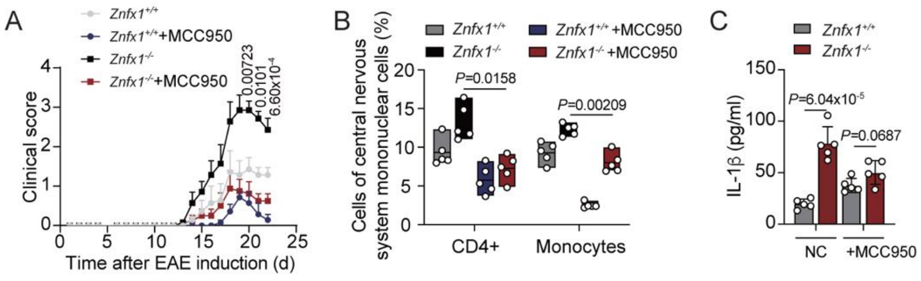

Appendix Figure S5. MCC950 alleviates excess inflammation-induced symptoms in the EAE model. (**A**) Clinical score after EAE induction of WT and *Znfx1*$^{-/-}$ mice pretreated with or without MCC950. n = 8 for the *Znfx1*$^{-/-}$ + MCC950 treatment group, n= 7 mice for all other groups. (**B**) CD4+ T cells and monocytes cells were analyzed by FACS after EAE induction of WT and *Znfx1*$^{-/-}$ mice treated with or without MCC950. (**C**) IL-1β in serum was measured by ELISA after EAE induction of WT and *Znfx1*$^{-/-}$ mice treated with or without MCC950. Note, In the presence of MCC950, there is still an impact of ZNFX1 on monocyte infiltration in the central nervous system and a small impact of ZNFX1 deficiency on EAE clinical scores and IL-1β. These results suggest that EAE may induce additional severity independent of NLRP3. Given that ZNFX1 is an RNA helicase, it may regulate mRNA stability through the yet-to-be-determined mechanism, in addition to acting as a virus sensor and NLRP3 inhibitor. Indeed, mRNA stability of some ISG genes such as OAS1, OAS2, IFIT1, and IFIT2 is affected by the loss of ZNFX1(Vavassori *et al.*, 2021).

Appendix Table S1 Primers for qPCR analysis

Target gene	Primer sequence(5'-3')
Human *IL-1β*	AGCTACGAATCTCCGACCAC
	CGTTATCCCATGTGTCGAAGAA
Human *TNF-α*	GAGGCCAAGCCCTGGTATG
	CGGGCCGATTGATCTCAGC
Human *ZNFX1*	AAATCAGGCCAATAACCCACC
	GGCCCTAAATCTCTCTTCCCT
Human *GAPDH*	GGAGCGAGATCCCTCCAAAAT
	GGCTGTTGTCATACTTCTCATGG
