# Supplementary material for: The human disease-associated gene ZNFX1 controls inflammation through inhibition of the NLRP3 inflammasome
Source: EMBO J. 2024 Sep 27;43(22):9. doi: 10.1038/s44318-024-00236-9 (PMC11574294; doi:10.1038/s44318-024-00236-9)
Supplement: Supplementary file 6 — Movie EV4 [file 44318_2024_236_MOESM6_ESM.zip › Movie EV4/Movie EV4 legends_final_submission_V2.docx]

**Movies EV4. Time-lapse imaging of GFP-ZNFX1, mCherry-NLRP3, and mMaroon1-TGN46 during NLRP3 inflammasome activation.** HeLa cells stably expressing GFP-ZNFX1, mCherry-NLRP3, and mMaroon1-TGN46 were grown in a 35mm confocal petri dish with a high-transparency borosilicate glass. The cells were treated with 10 μM Nigericin and imaged every minute for 120 minutes in a 37℃ chamber with 5% CO2, using a NIKON ECLIPSE Ti2-E with a CSU-W1 spinning disc and a Plan Apochromat 100× NA 1.45 oil objective. Time is expressed as hh:mm.
