# Supplementary figures and images for: The human disease-associated gene ZNFX1 controls inflammation through inhibition of the NLRP3 inflammasome
Source: EMBO J. 2024 Sep 27;43(22):9. doi: 10.1038/s44318-024-00236-9 (PMC11574294; doi:10.1038/s44318-024-00236-9)

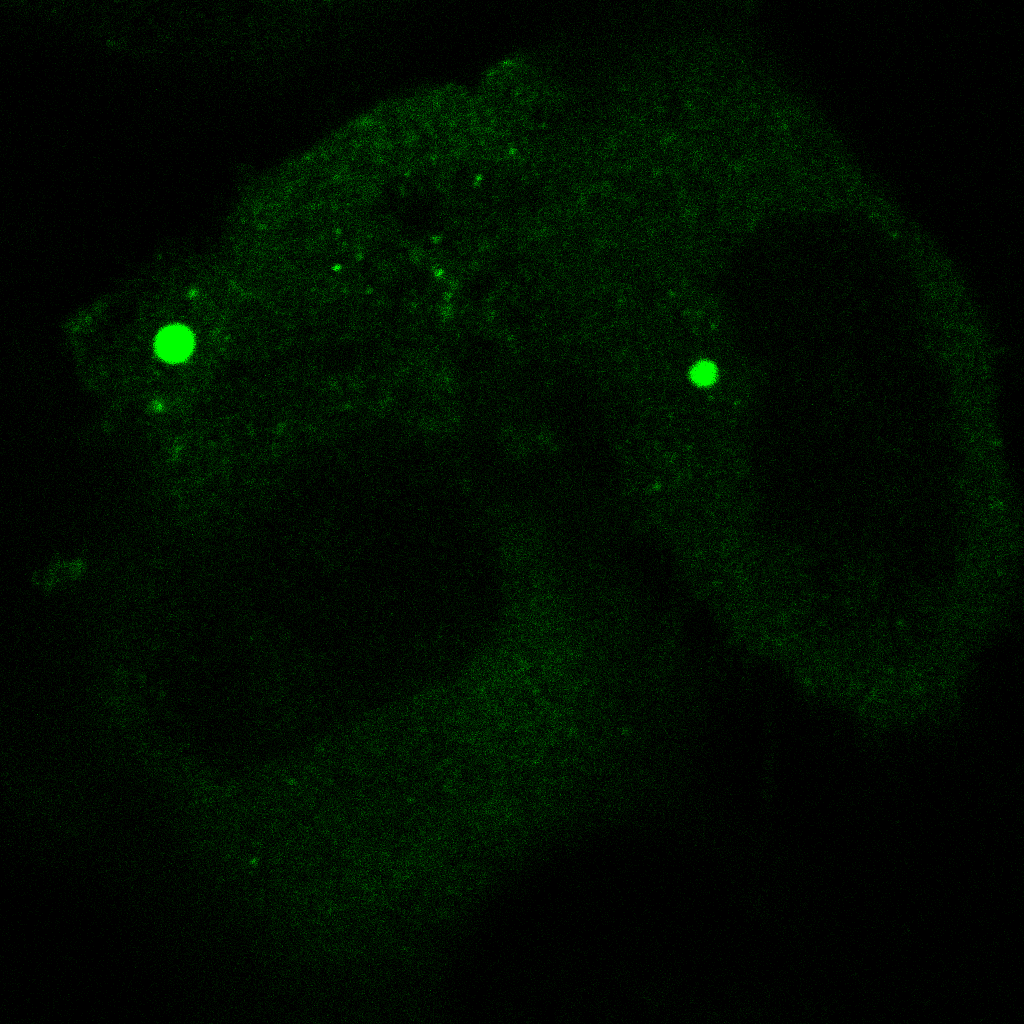

Supplement: Supplementary file 10 — Source data Fig. 4 [file 44318_2024_236_MOESM10_ESM.zip › Figure 4_final_submission_V3/Figure 4E/KO1 ZNFX1-GFP.tif]

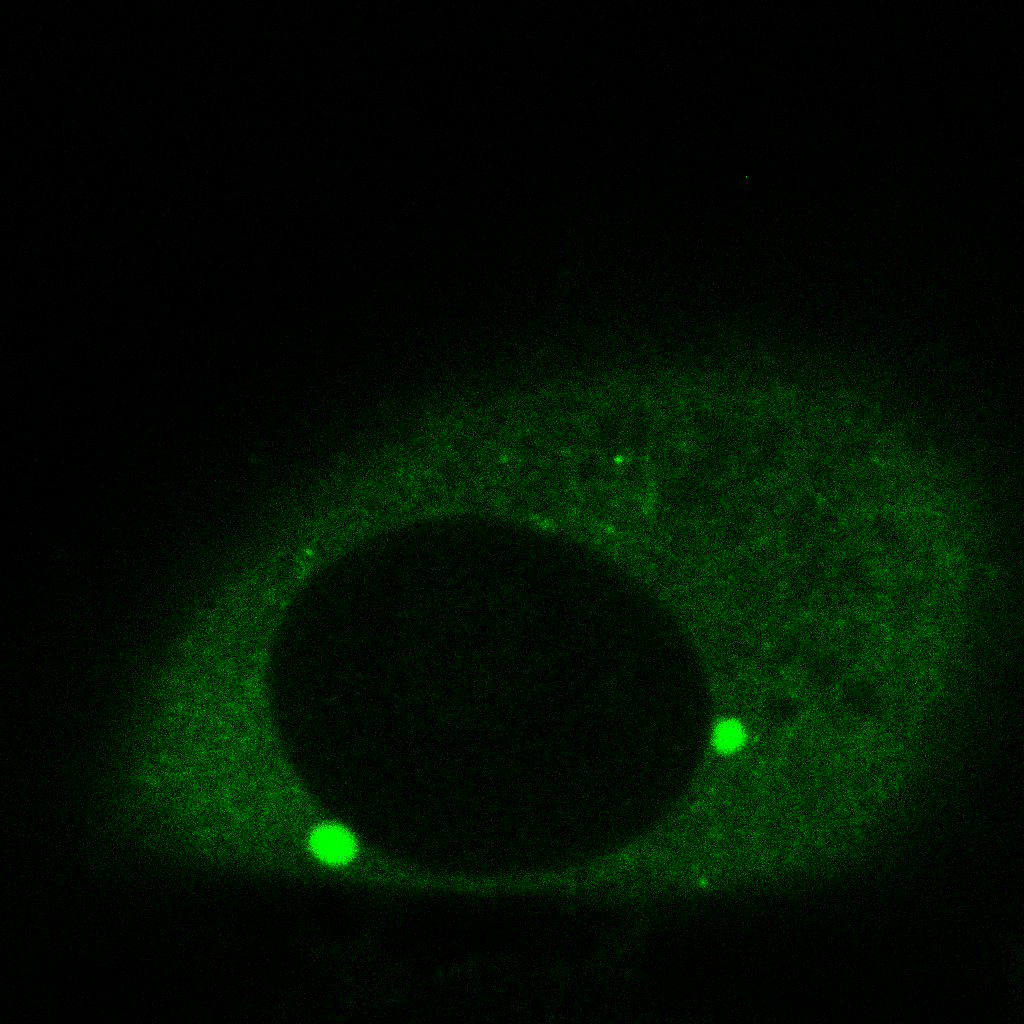

Supplement: Supplementary file 10 — Source data Fig. 4 [file 44318_2024_236_MOESM10_ESM.zip › Figure 4_final_submission_V3/Figure 4E/KO2 ZNFX1-GFP.tif]

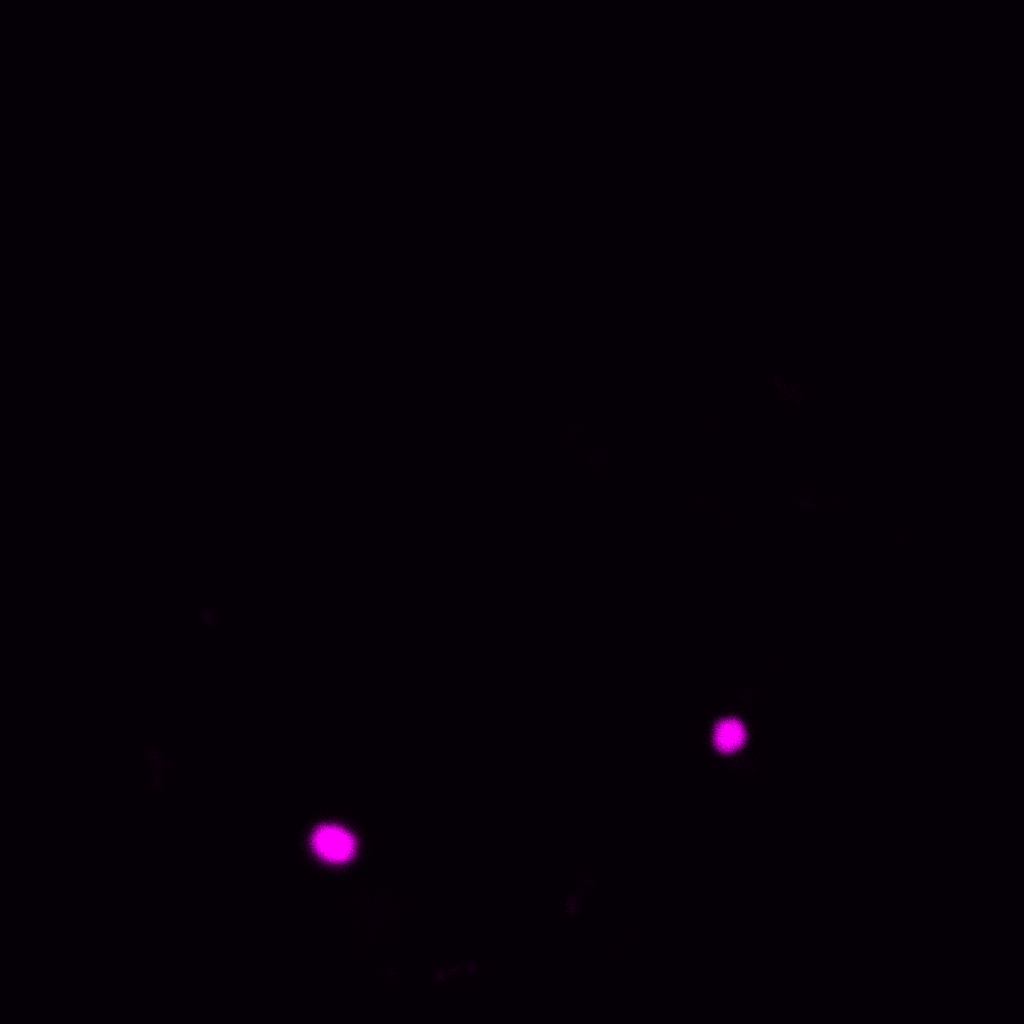

Supplement: Supplementary file 10 — Source data Fig. 4 [file 44318_2024_236_MOESM10_ESM.zip › Figure 4_final_submission_V3/Figure 4E/KO2 mMaroon1-ASC.tif]

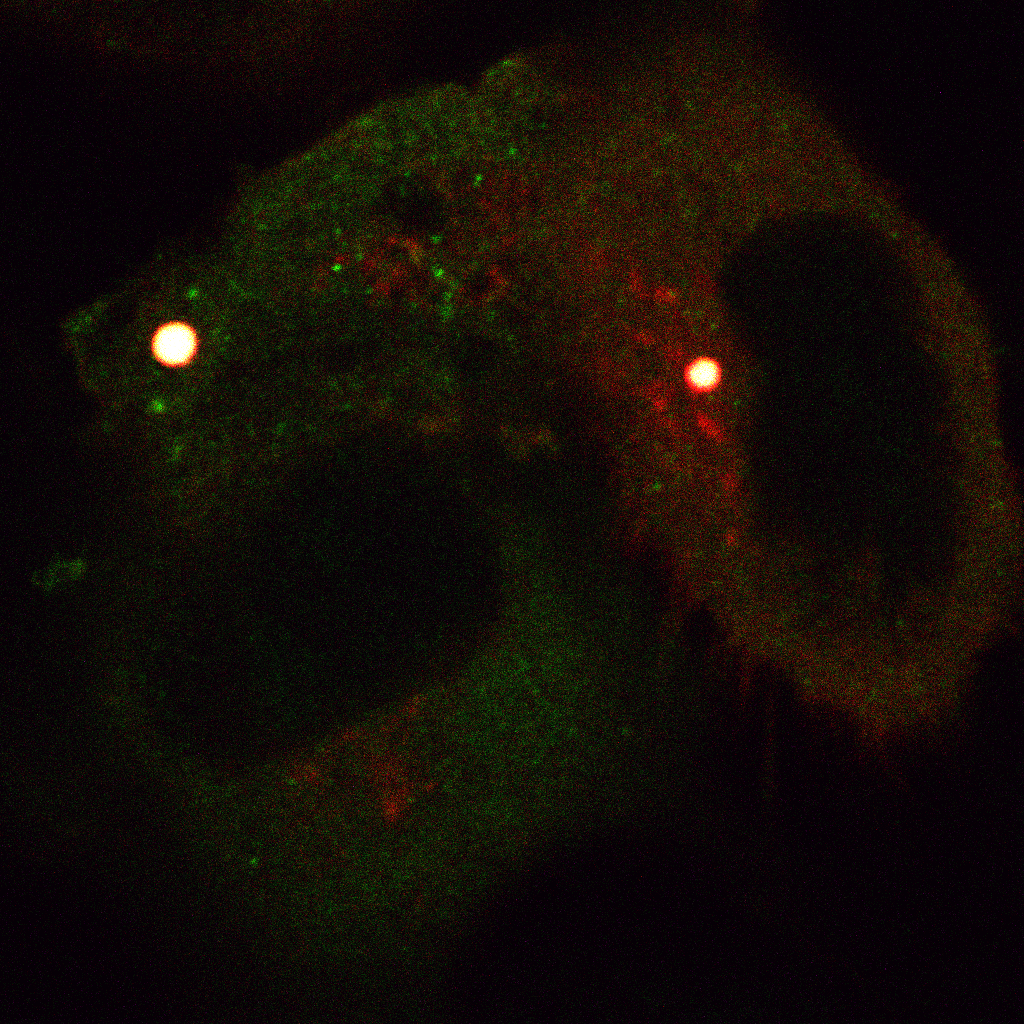

Supplement: Supplementary file 10 — Source data Fig. 4 [file 44318_2024_236_MOESM10_ESM.zip › Figure 4_final_submission_V3/Figure 4E/KO1 Merge.tif]

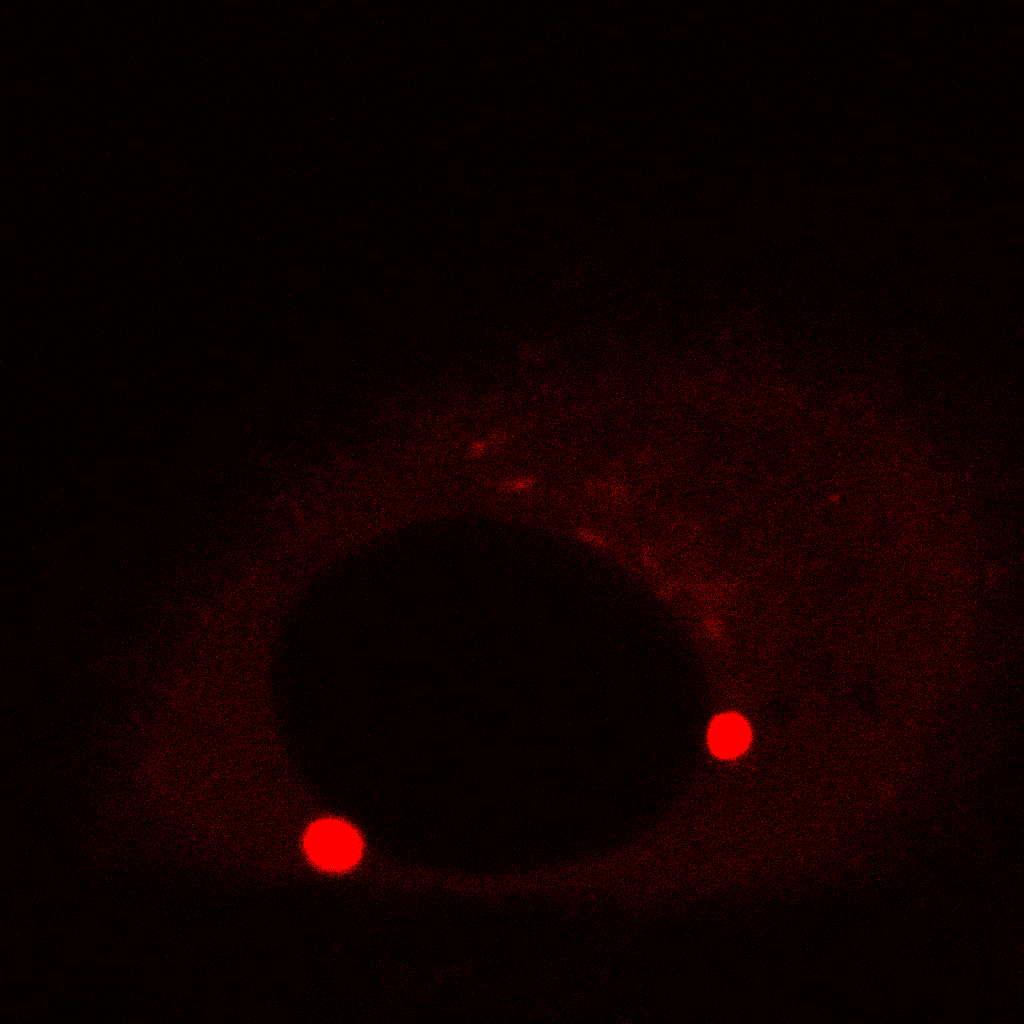

Supplement: Supplementary file 10 — Source data Fig. 4 [file 44318_2024_236_MOESM10_ESM.zip › Figure 4_final_submission_V3/Figure 4E/KO2 NLRP3-mCherry.tif]

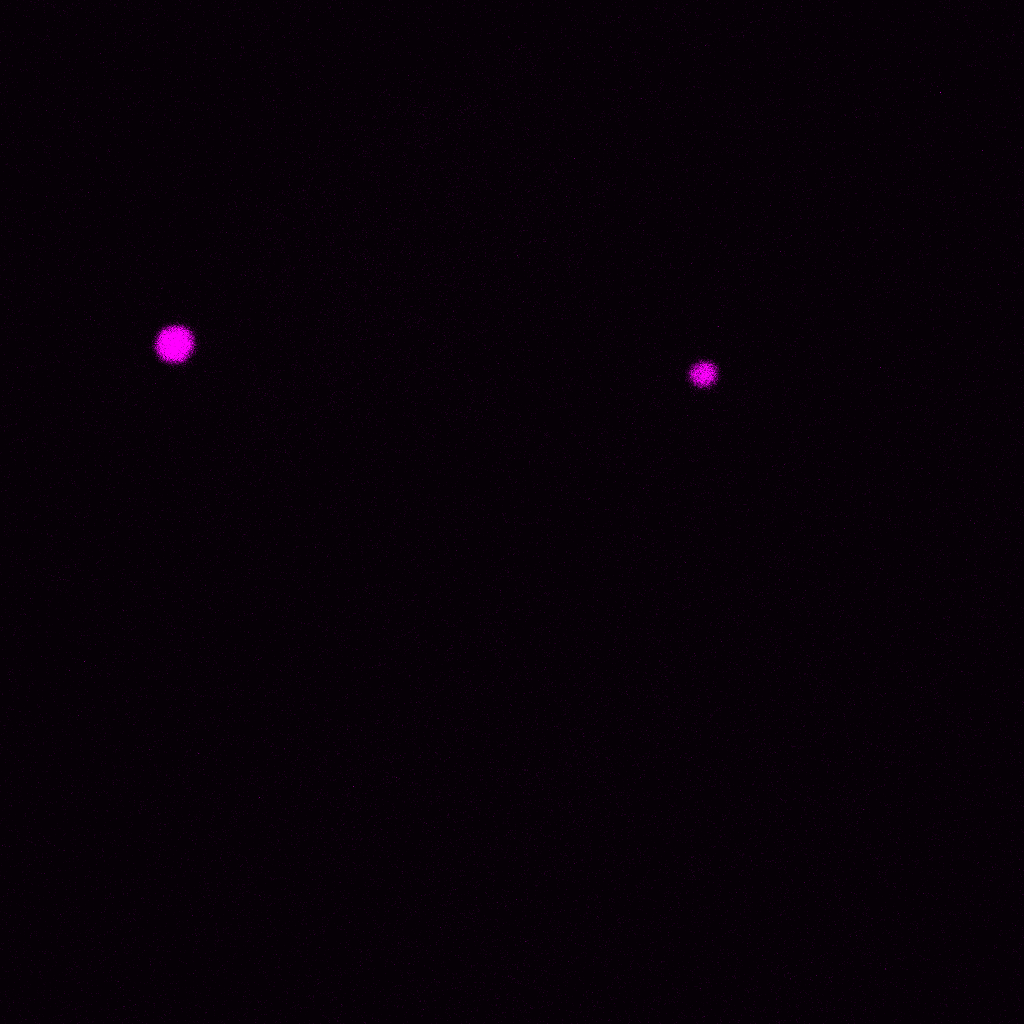

Supplement: Supplementary file 10 — Source data Fig. 4 [file 44318_2024_236_MOESM10_ESM.zip › Figure 4_final_submission_V3/Figure 4E/KO1 mMaroon1-ASC.tif]

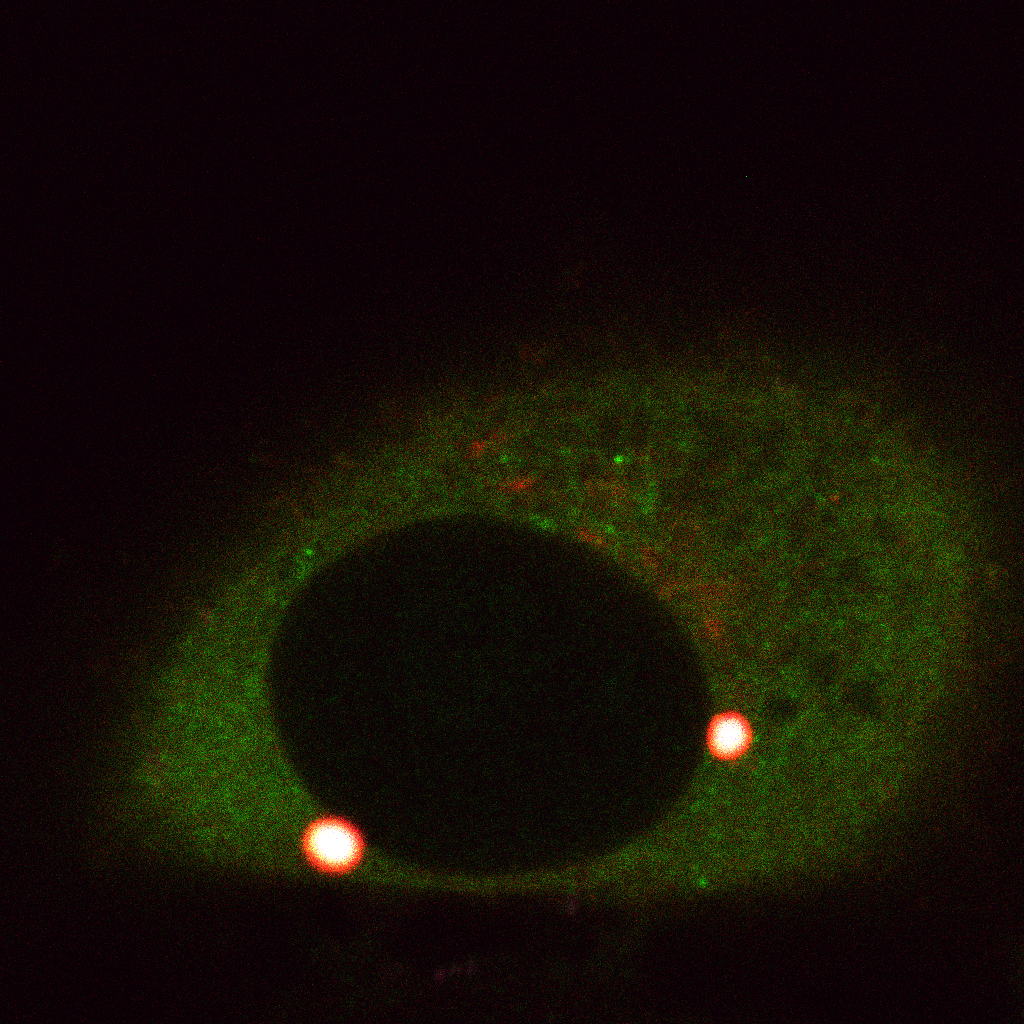

Supplement: Supplementary file 10 — Source data Fig. 4 [file 44318_2024_236_MOESM10_ESM.zip › Figure 4_final_submission_V3/Figure 4E/KO2 Merge .tif]

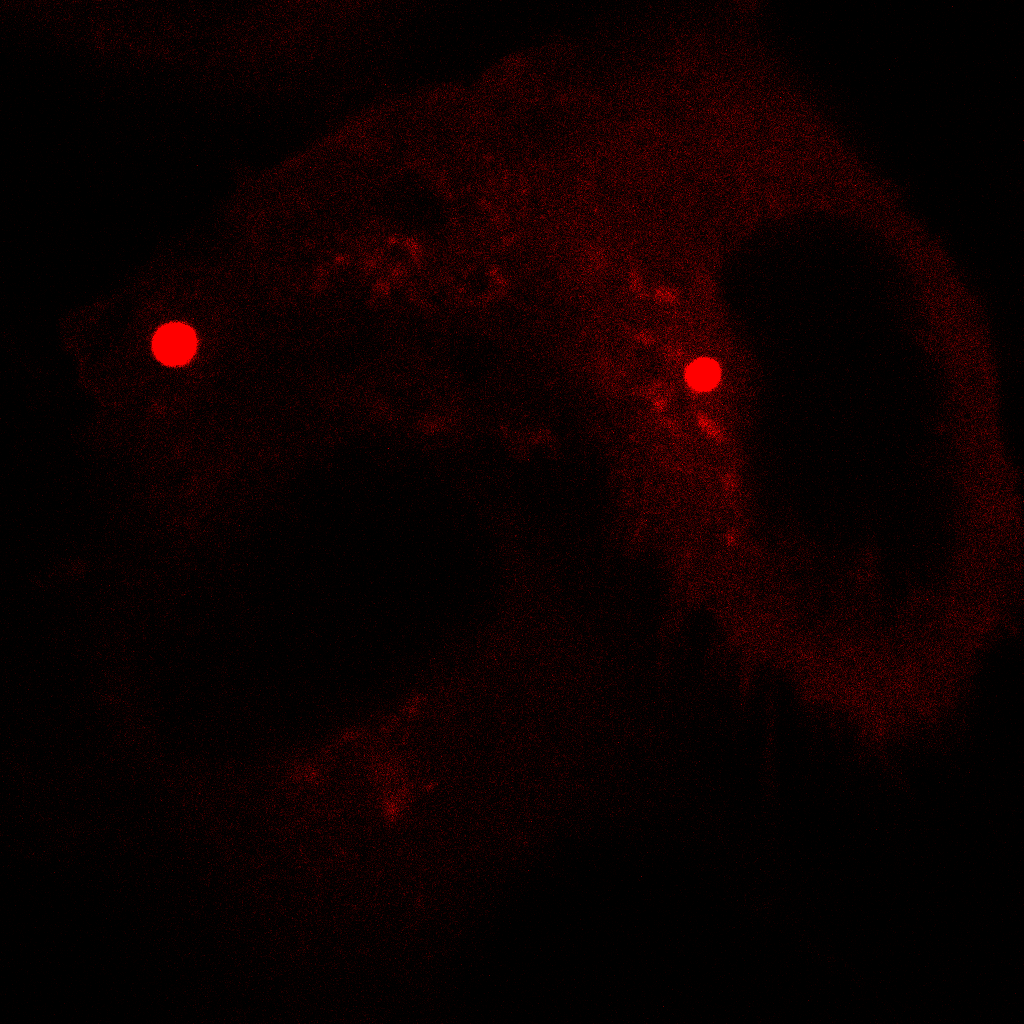

Supplement: Supplementary file 10 — Source data Fig. 4 [file 44318_2024_236_MOESM10_ESM.zip › Figure 4_final_submission_V3/Figure 4E/KO1 NLRP3-mCherry.tif]

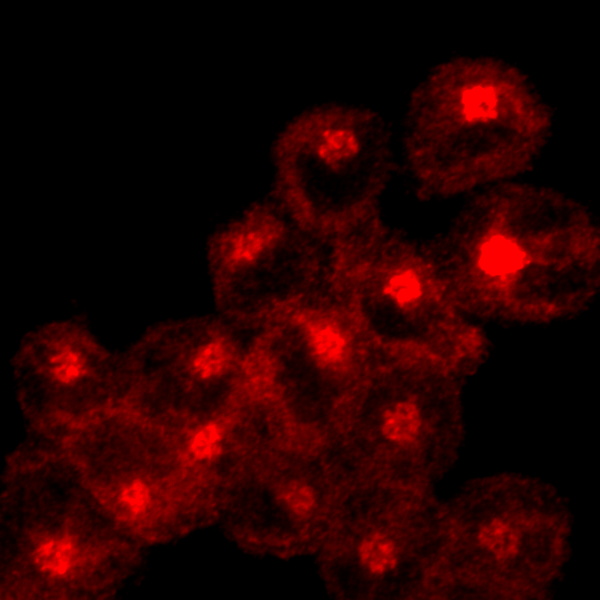

Supplement: Supplementary file 10 — Source data Fig. 4 [file 44318_2024_236_MOESM10_ESM.zip › Figure 4_final_submission_V3/Figure 4C/KO NLRP3.tif]

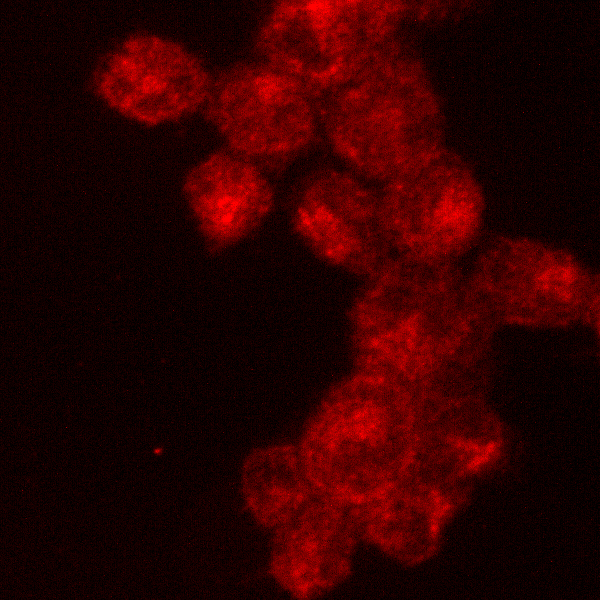

Supplement: Supplementary file 10 — Source data Fig. 4 [file 44318_2024_236_MOESM10_ESM.zip › Figure 4_final_submission_V3/Figure 4C/RE NLRP3.tif]

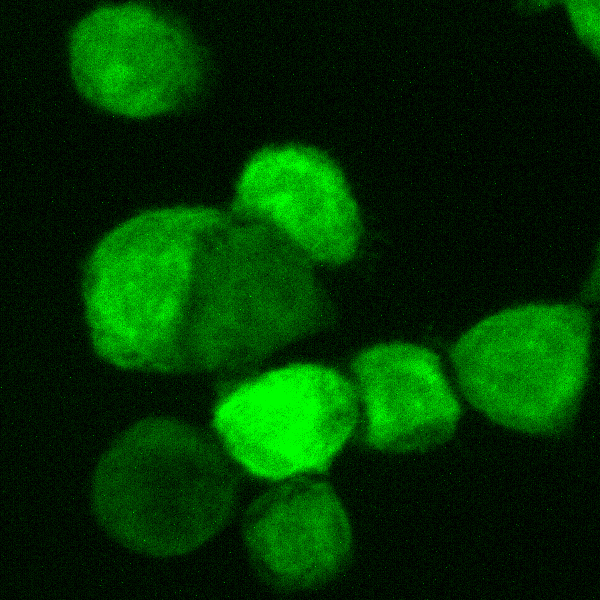

Supplement: Supplementary file 10 — Source data Fig. 4 [file 44318_2024_236_MOESM10_ESM.zip › Figure 4_final_submission_V3/Figure 4C/OE ZNFX1-GFP.tif]

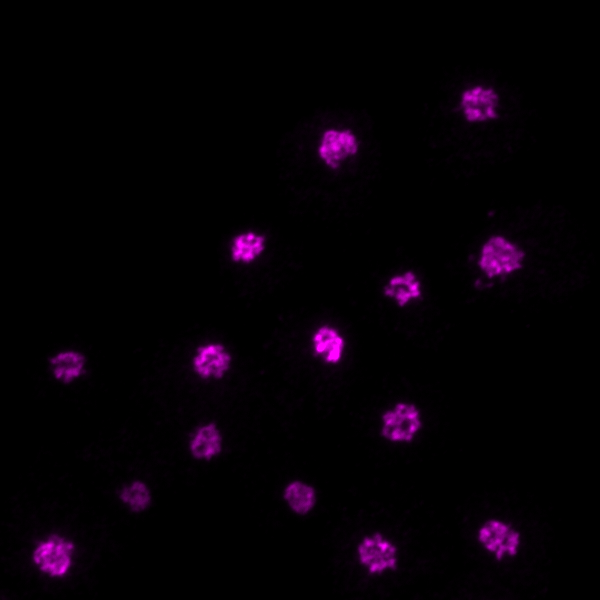

Supplement: Supplementary file 10 — Source data Fig. 4 [file 44318_2024_236_MOESM10_ESM.zip › Figure 4_final_submission_V3/Figure 4C/KO TGN46.tif]

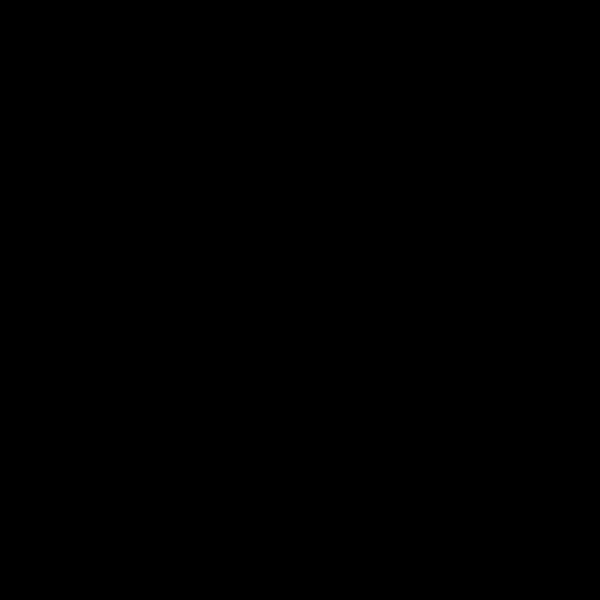

Supplement: Supplementary file 10 — Source data Fig. 4 [file 44318_2024_236_MOESM10_ESM.zip › Figure 4_final_submission_V3/Figure 4C/KO ZNFX1-GFP.tif]

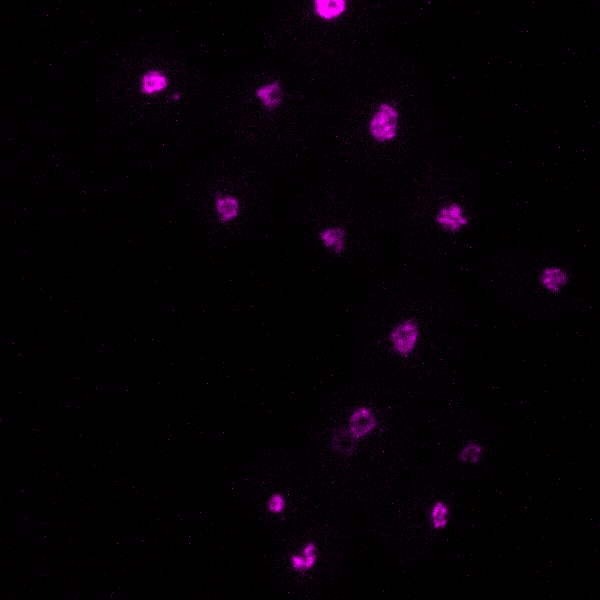

Supplement: Supplementary file 10 — Source data Fig. 4 [file 44318_2024_236_MOESM10_ESM.zip › Figure 4_final_submission_V3/Figure 4C/RE TGN46.tif]

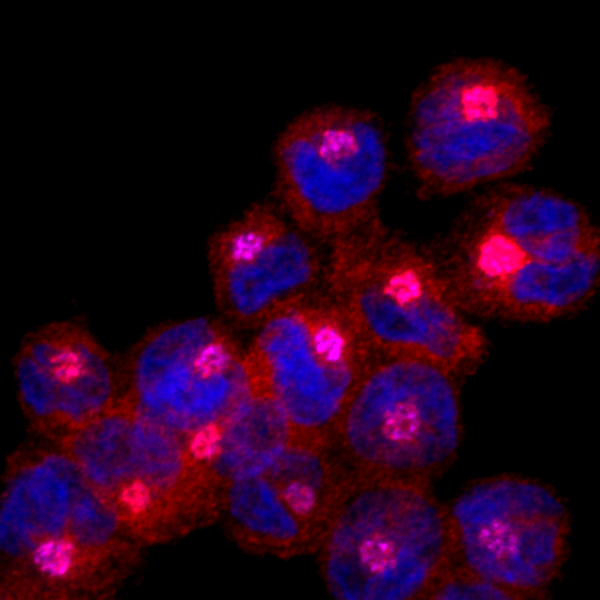

Supplement: Supplementary file 10 — Source data Fig. 4 [file 44318_2024_236_MOESM10_ESM.zip › Figure 4_final_submission_V3/Figure 4C/KO Merge.tif]

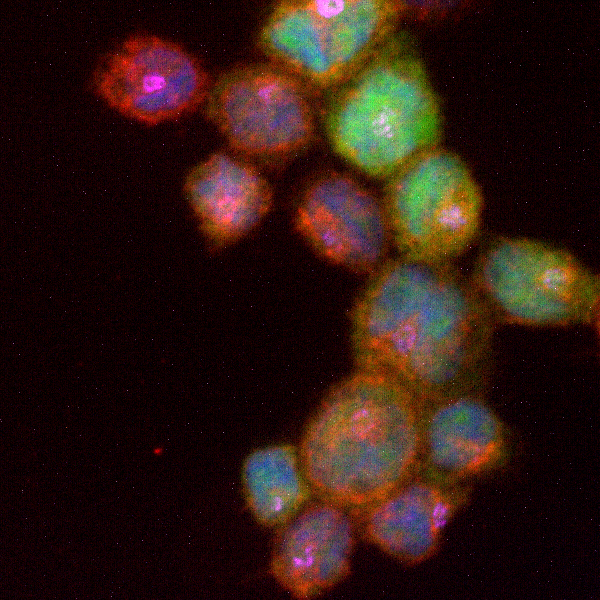

Supplement: Supplementary file 10 — Source data Fig. 4 [file 44318_2024_236_MOESM10_ESM.zip › Figure 4_final_submission_V3/Figure 4C/RE Merge.tif]

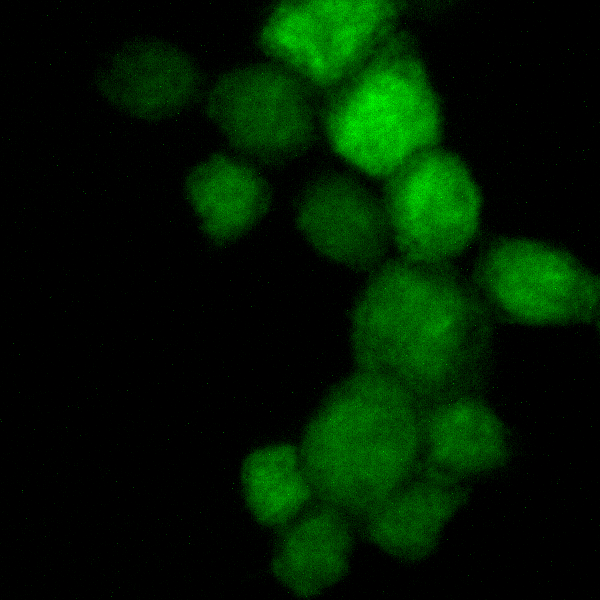

Supplement: Supplementary file 10 — Source data Fig. 4 [file 44318_2024_236_MOESM10_ESM.zip › Figure 4_final_submission_V3/Figure 4C/RE ZNFX1-GFP.tif]

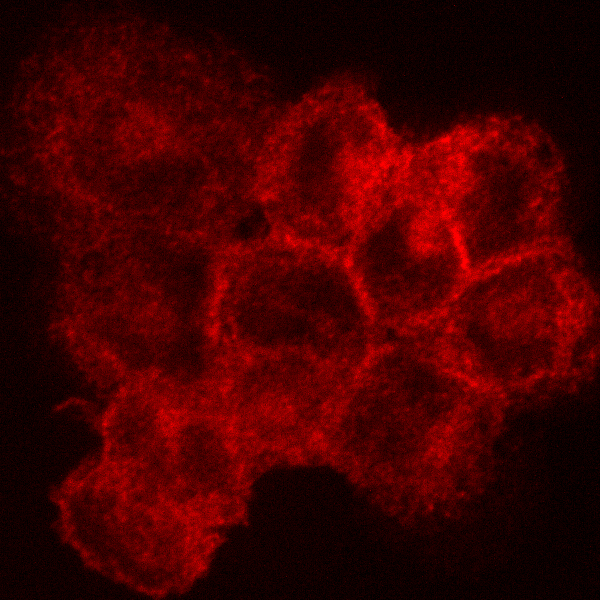

Supplement: Supplementary file 10 — Source data Fig. 4 [file 44318_2024_236_MOESM10_ESM.zip › Figure 4_final_submission_V3/Figure 4C/WT NLRP3.tif]

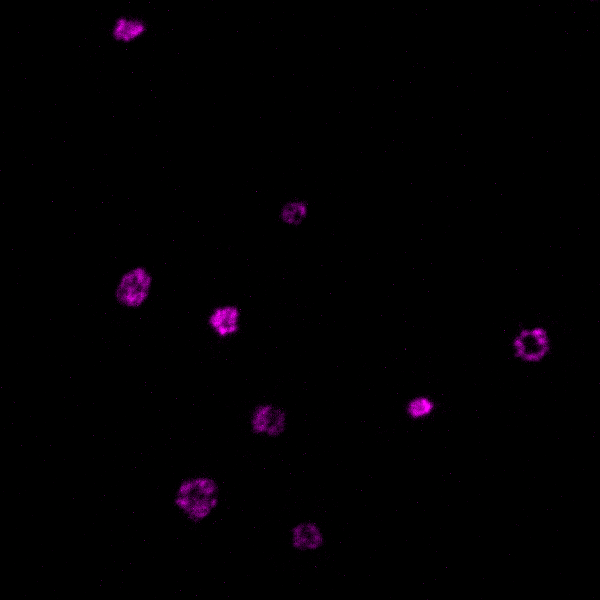

Supplement: Supplementary file 10 — Source data Fig. 4 [file 44318_2024_236_MOESM10_ESM.zip › Figure 4_final_submission_V3/Figure 4C/OE TGN46.tif]

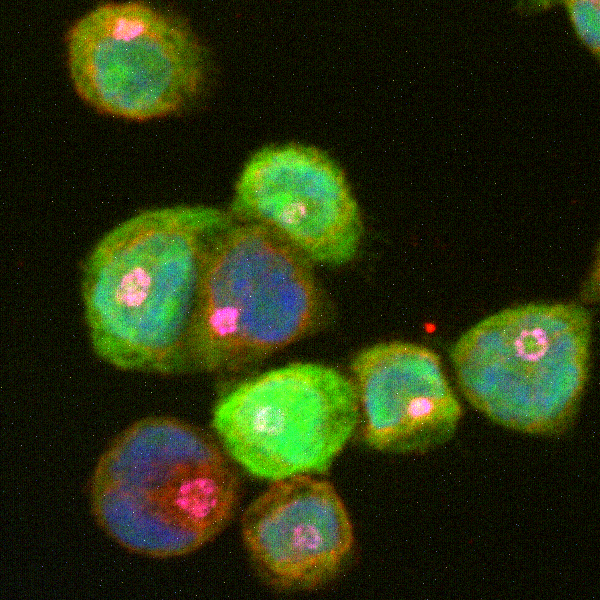

Supplement: Supplementary file 10 — Source data Fig. 4 [file 44318_2024_236_MOESM10_ESM.zip › Figure 4_final_submission_V3/Figure 4C/OE Merge.tif]

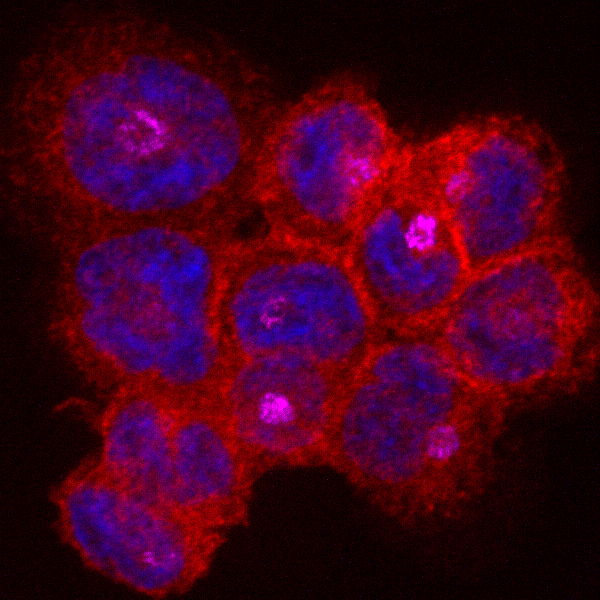

Supplement: Supplementary file 10 — Source data Fig. 4 [file 44318_2024_236_MOESM10_ESM.zip › Figure 4_final_submission_V3/Figure 4C/WT Merge.tif]

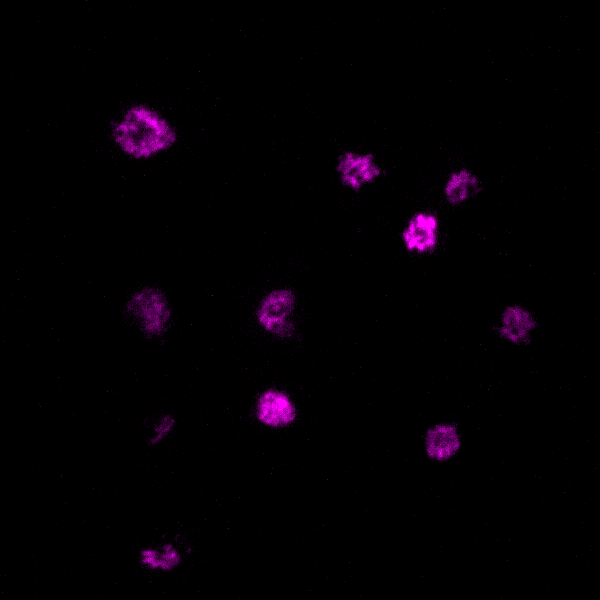

Supplement: Supplementary file 10 — Source data Fig. 4 [file 44318_2024_236_MOESM10_ESM.zip › Figure 4_final_submission_V3/Figure 4C/WT TGN46.tif]

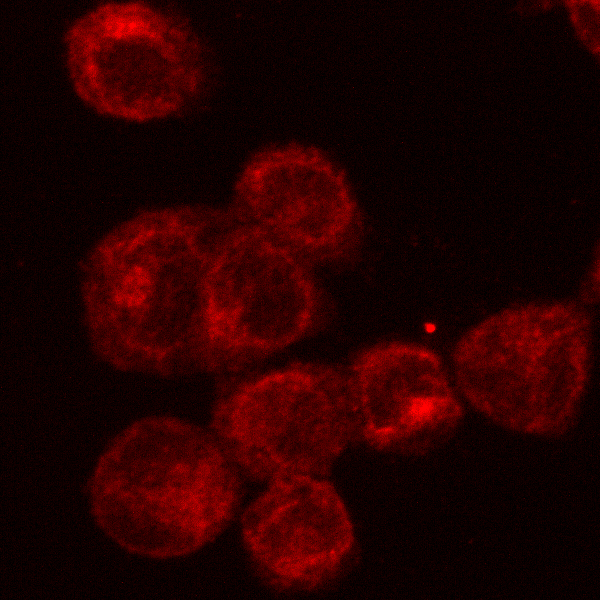

Supplement: Supplementary file 10 — Source data Fig. 4 [file 44318_2024_236_MOESM10_ESM.zip › Figure 4_final_submission_V3/Figure 4C/OE NLRP3.tif]

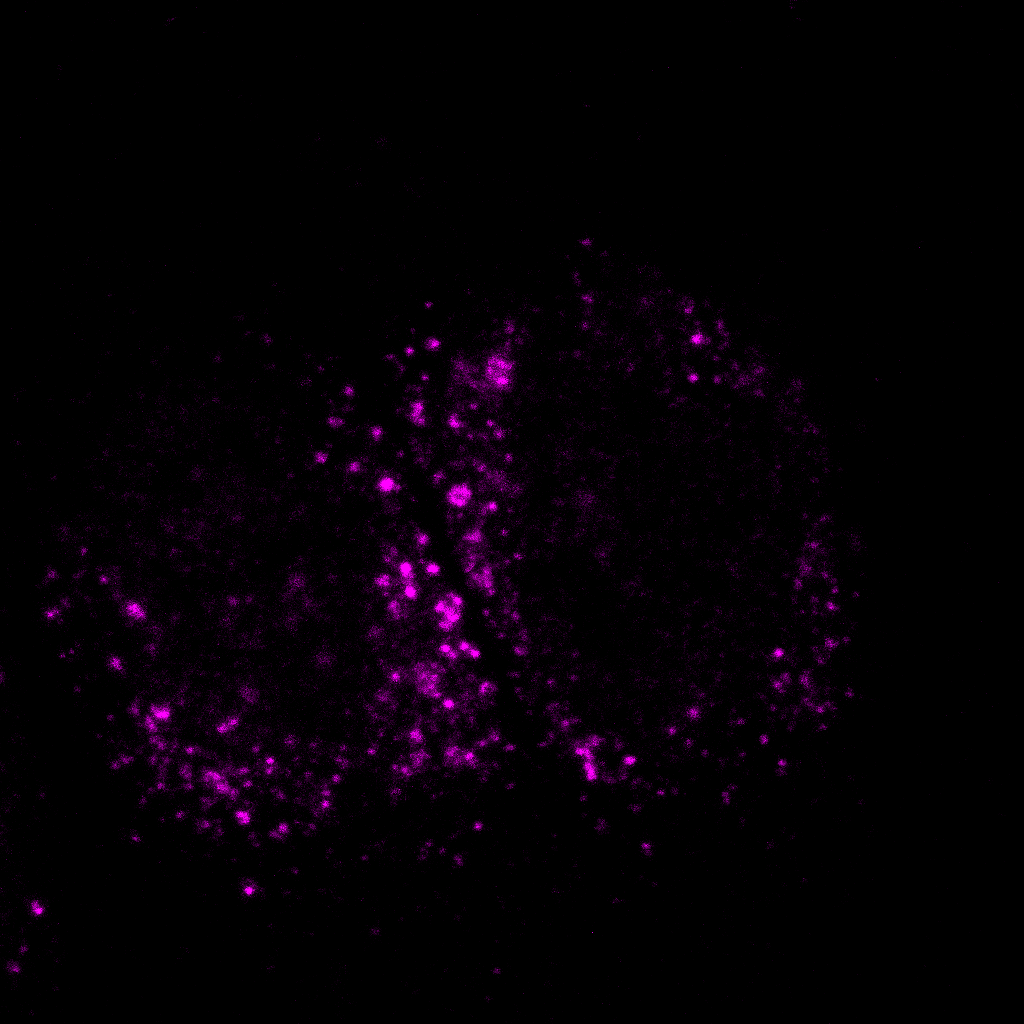

Supplement: Supplementary file 10 — Source data Fig. 4 [file 44318_2024_236_MOESM10_ESM.zip › Figure 4_final_submission_V3/Figure 4A/Nig 60 TGN46.tif]

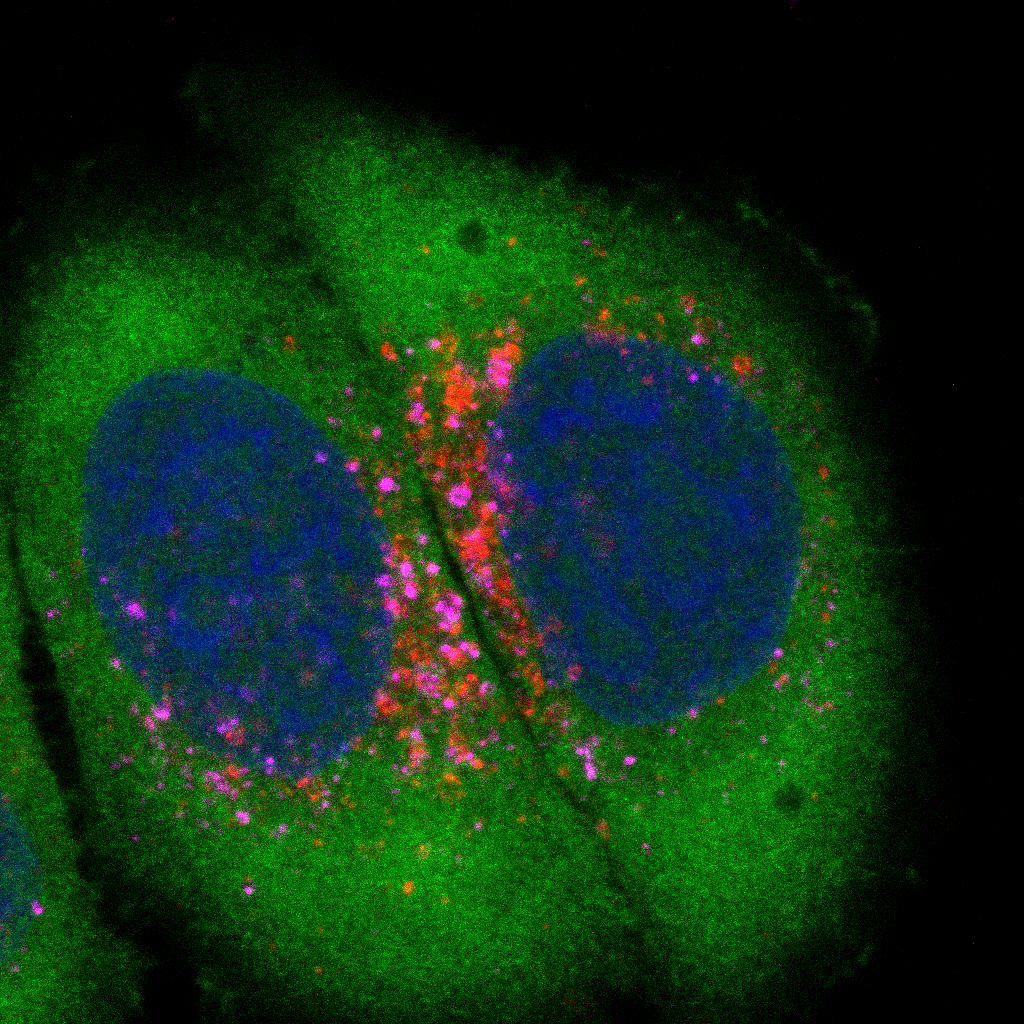

Supplement: Supplementary file 10 — Source data Fig. 4 [file 44318_2024_236_MOESM10_ESM.zip › Figure 4_final_submission_V3/Figure 4A/Nig 60 Merge.tif]

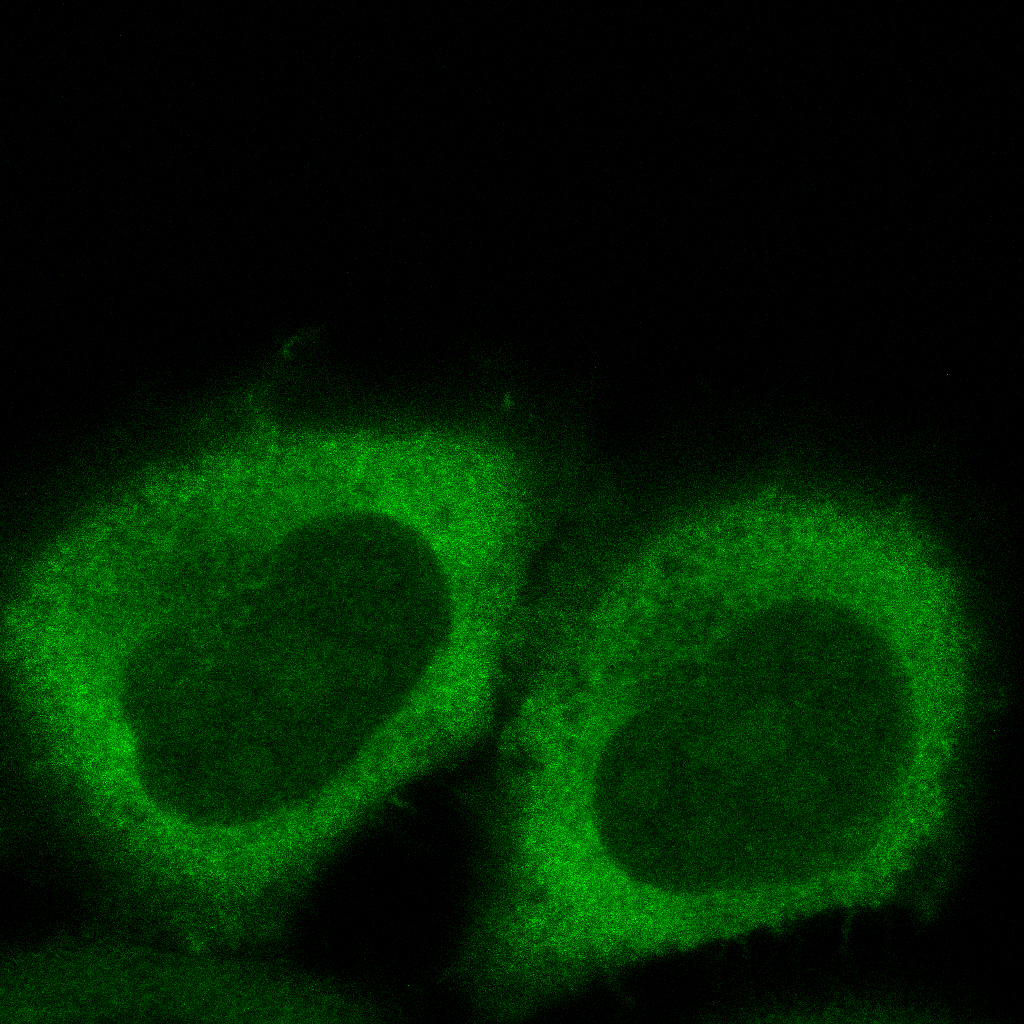

Supplement: Supplementary file 10 — Source data Fig. 4 [file 44318_2024_236_MOESM10_ESM.zip › Figure 4_final_submission_V3/Figure 4A/Nig 30 ZNFX1-GFP.tif]

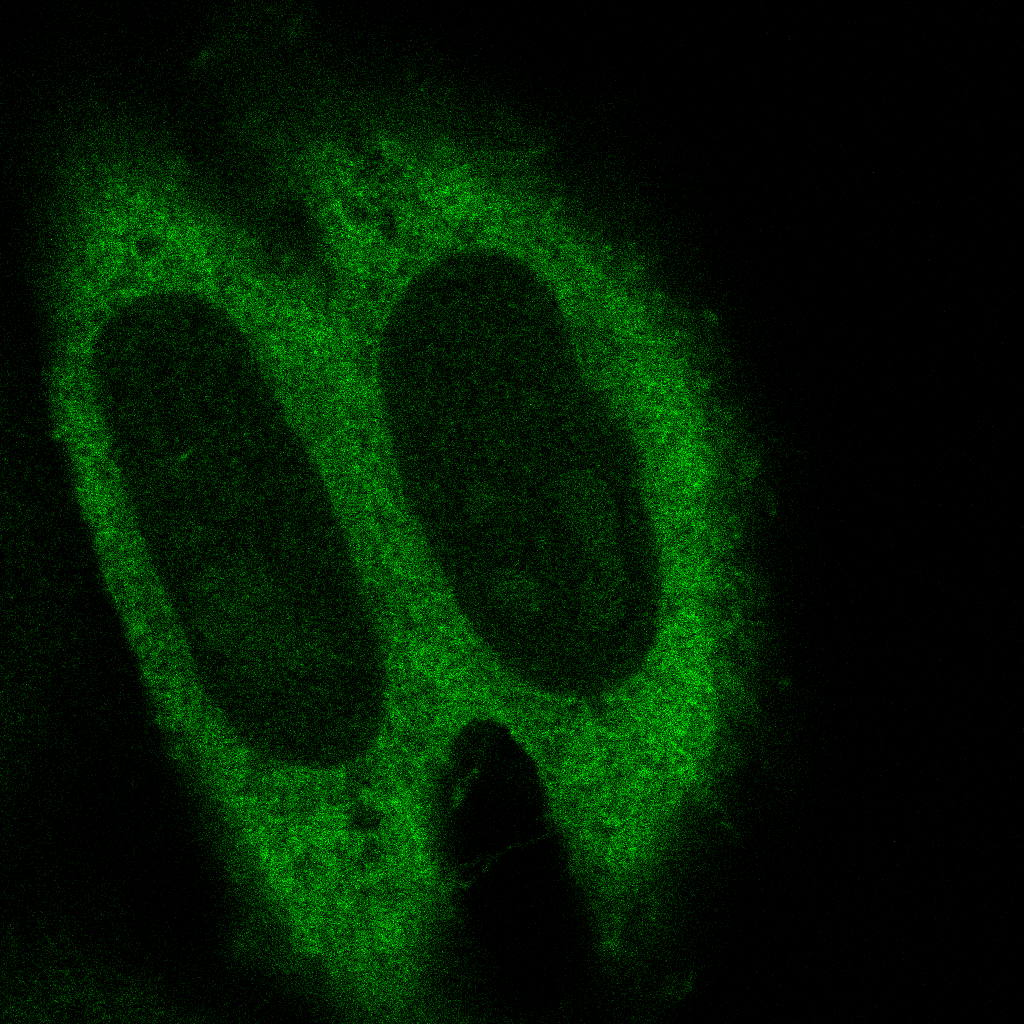

Supplement: Supplementary file 10 — Source data Fig. 4 [file 44318_2024_236_MOESM10_ESM.zip › Figure 4_final_submission_V3/Figure 4A/Mock ZNFX1-GFP.tif]

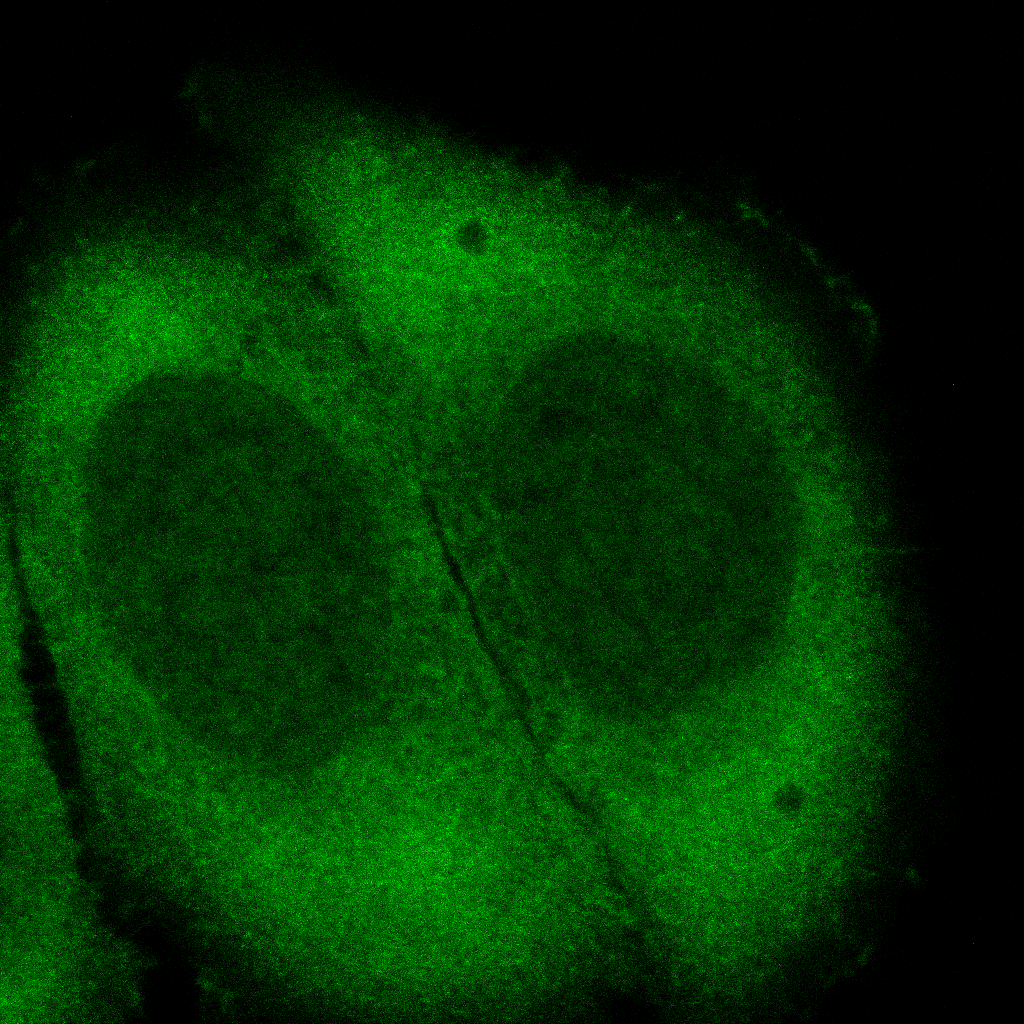

Supplement: Supplementary file 10 — Source data Fig. 4 [file 44318_2024_236_MOESM10_ESM.zip › Figure 4_final_submission_V3/Figure 4A/Nig 60 ZNFX1-GFP.tif]

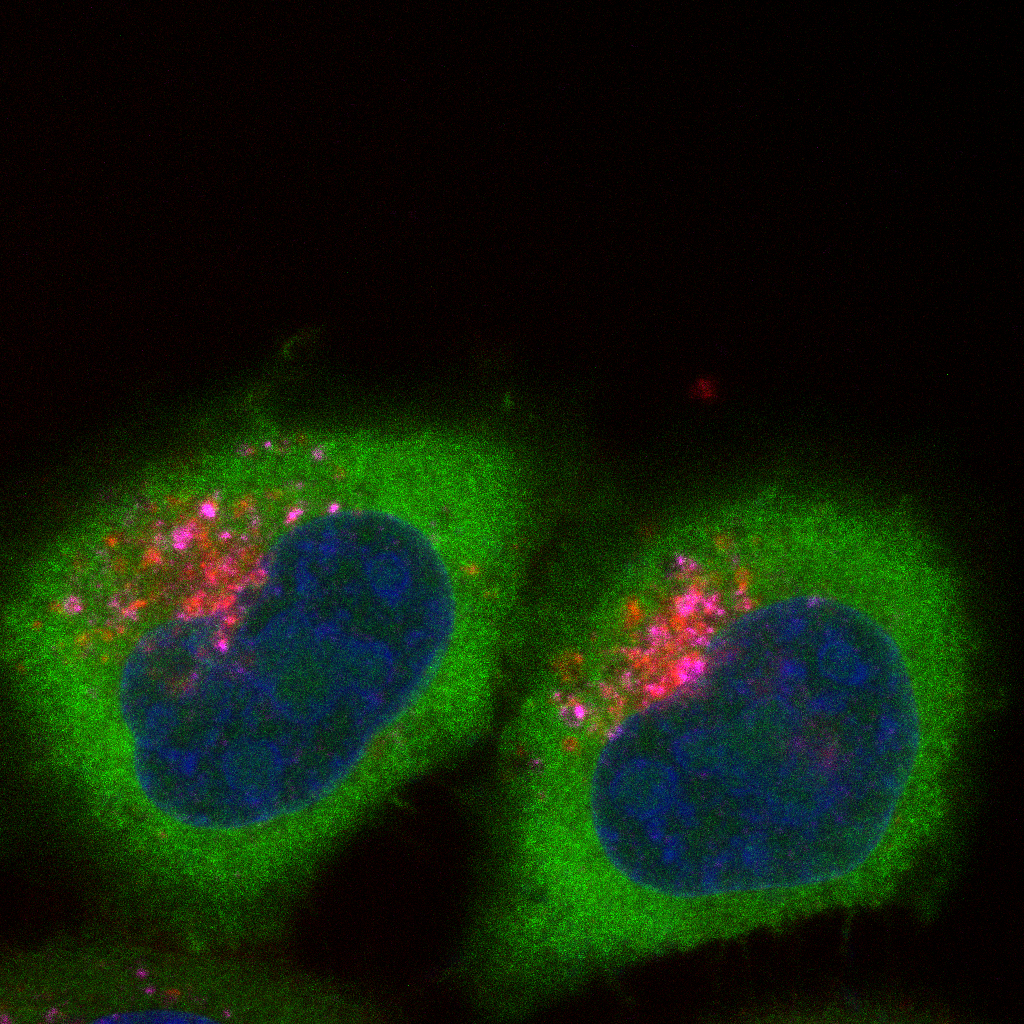

Supplement: Supplementary file 10 — Source data Fig. 4 [file 44318_2024_236_MOESM10_ESM.zip › Figure 4_final_submission_V3/Figure 4A/Nig 30 Merge .tif]

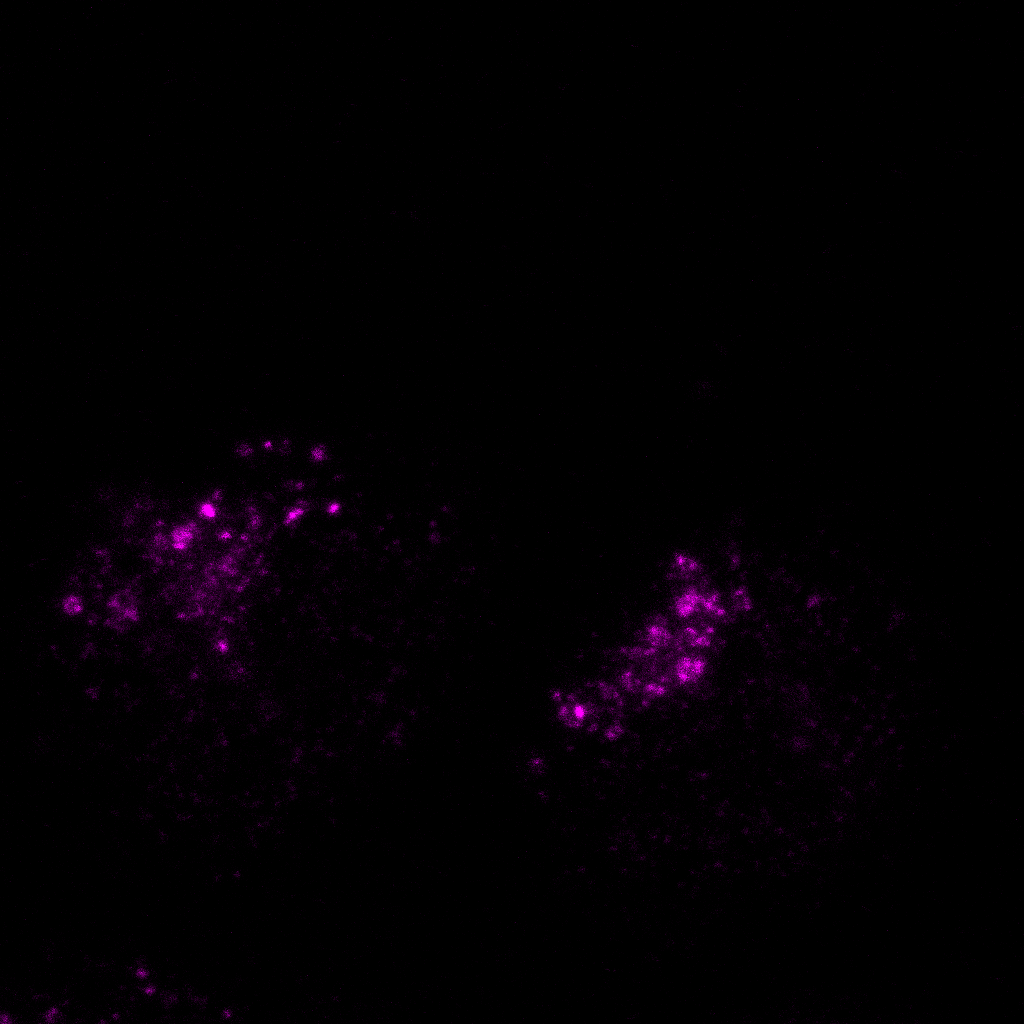

Supplement: Supplementary file 10 — Source data Fig. 4 [file 44318_2024_236_MOESM10_ESM.zip › Figure 4_final_submission_V3/Figure 4A/Nig 30 TGN36.tif]

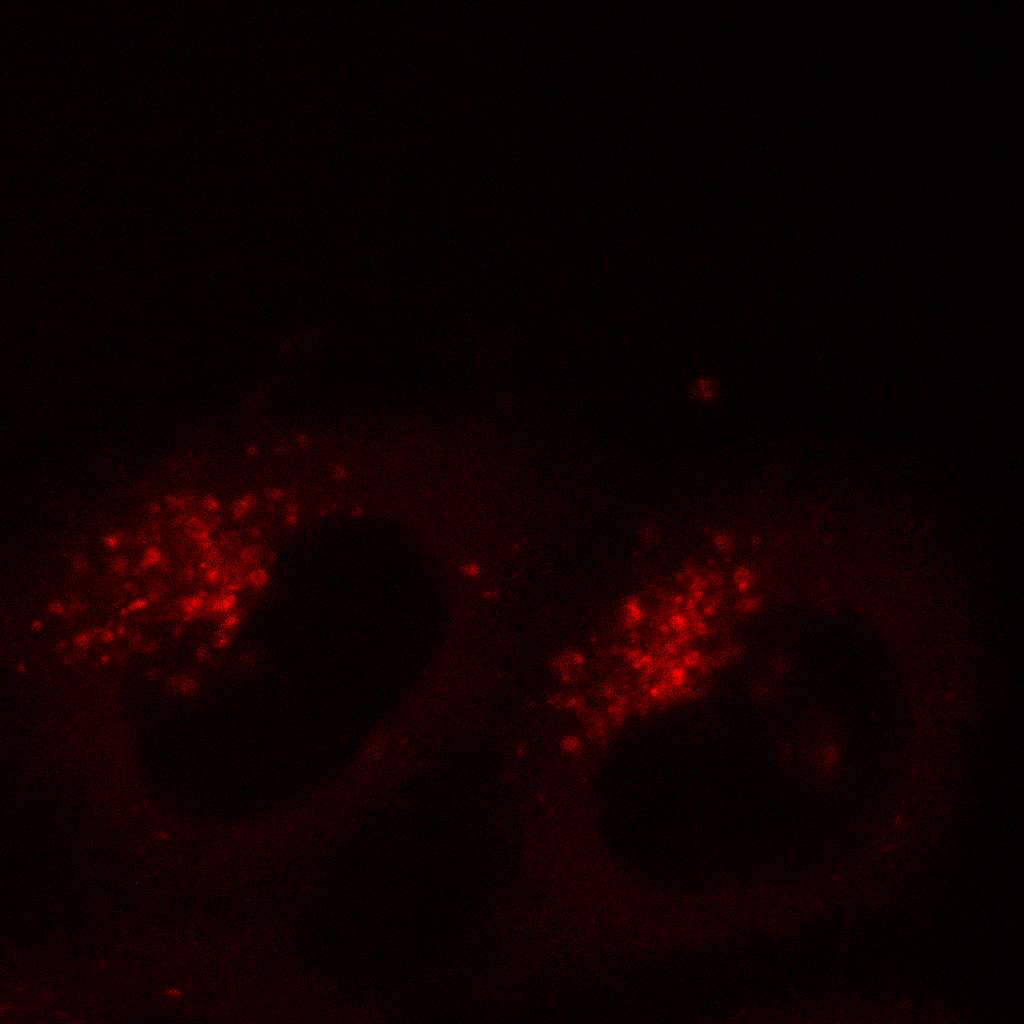

Supplement: Supplementary file 10 — Source data Fig. 4 [file 44318_2024_236_MOESM10_ESM.zip › Figure 4_final_submission_V3/Figure 4A/Nig 30 NLRP3-mCherry.tif]

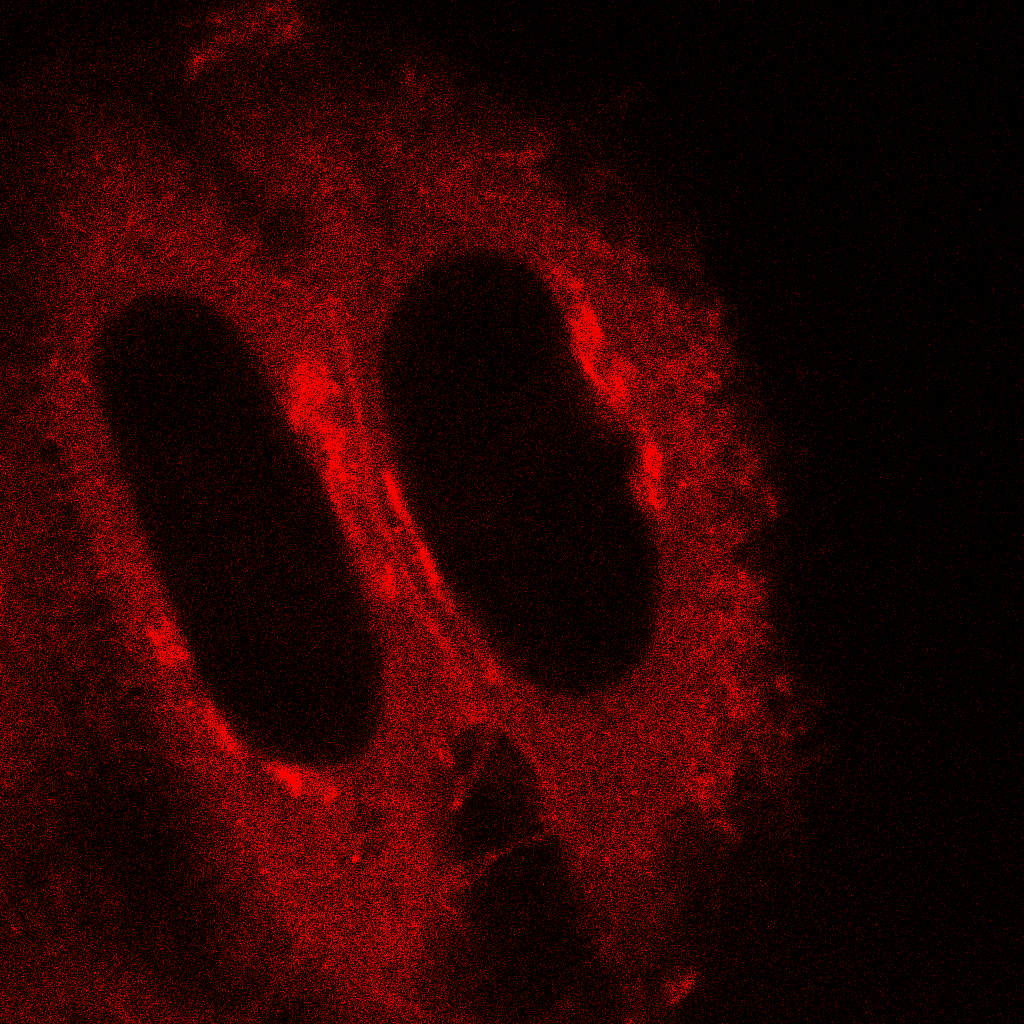

Supplement: Supplementary file 10 — Source data Fig. 4 [file 44318_2024_236_MOESM10_ESM.zip › Figure 4_final_submission_V3/Figure 4A/Mock NLRP3-mCherry.tif]

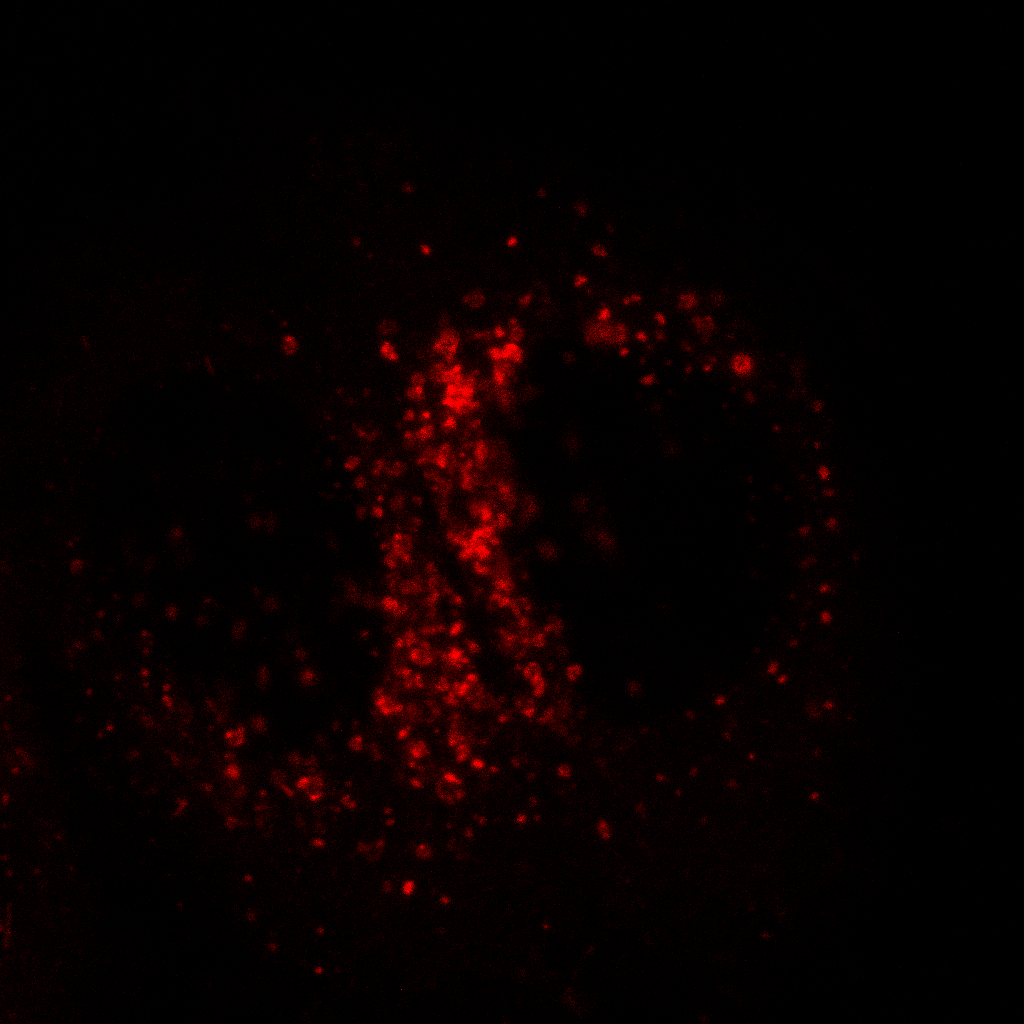

Supplement: Supplementary file 10 — Source data Fig. 4 [file 44318_2024_236_MOESM10_ESM.zip › Figure 4_final_submission_V3/Figure 4A/Nig 60 NLRP3-mCherry.tif]

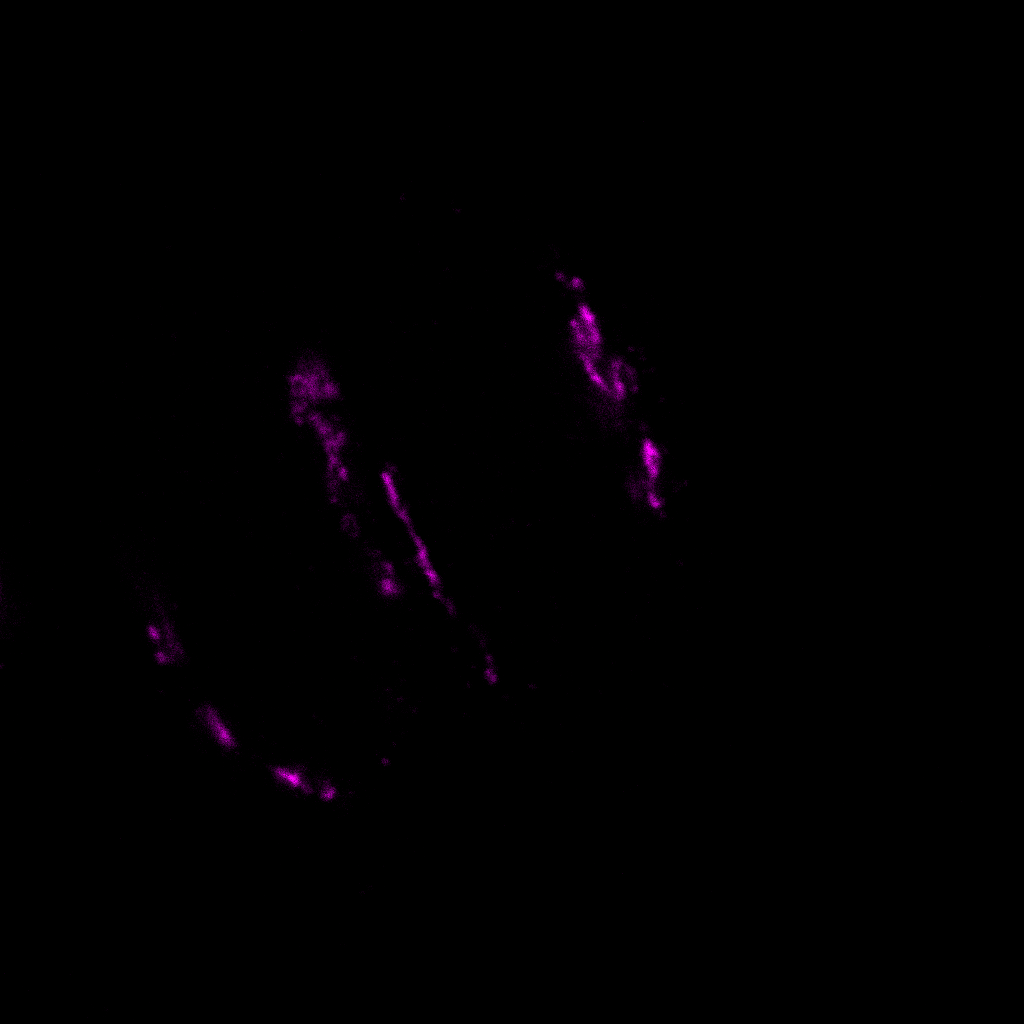

Supplement: Supplementary file 10 — Source data Fig. 4 [file 44318_2024_236_MOESM10_ESM.zip › Figure 4_final_submission_V3/Figure 4A/Mock TGN46.tif]

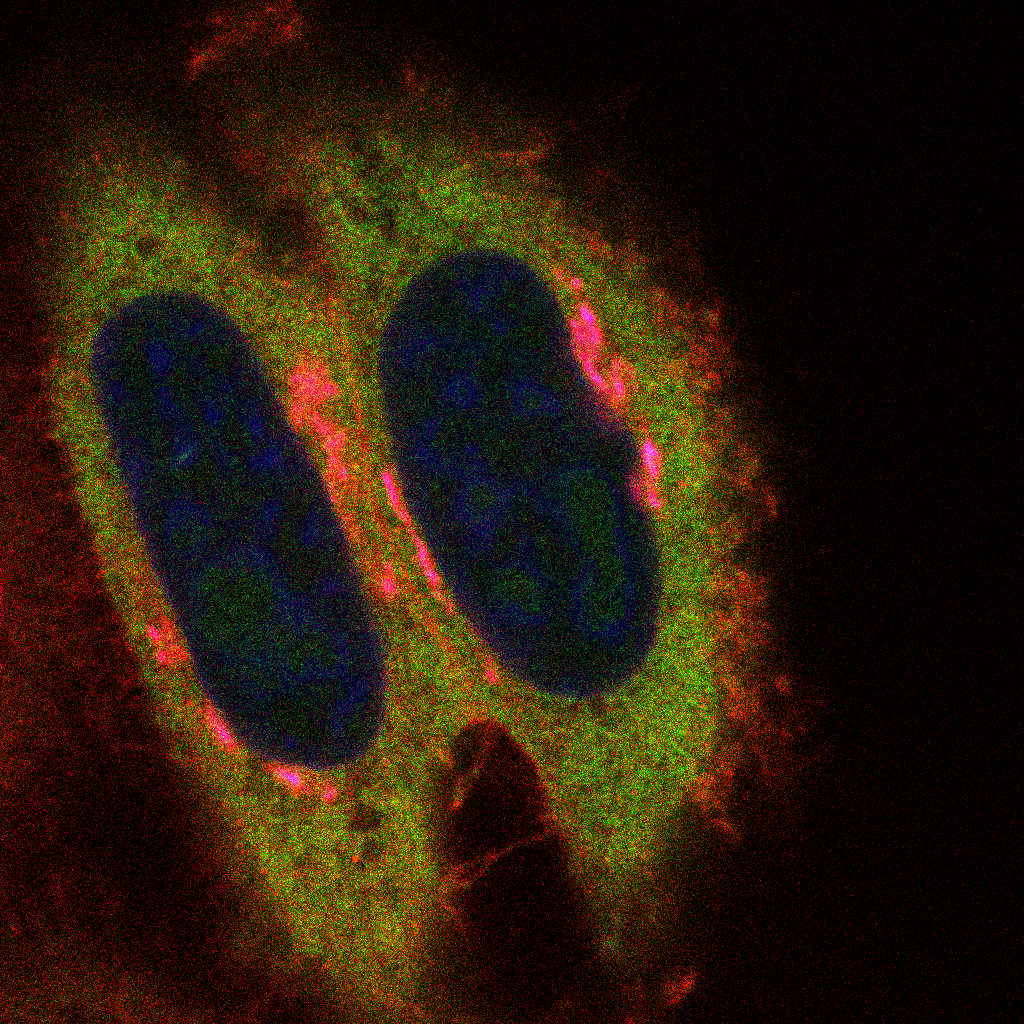

Supplement: Supplementary file 10 — Source data Fig. 4 [file 44318_2024_236_MOESM10_ESM.zip › Figure 4_final_submission_V3/Figure 4A/Mock Merge.tif]

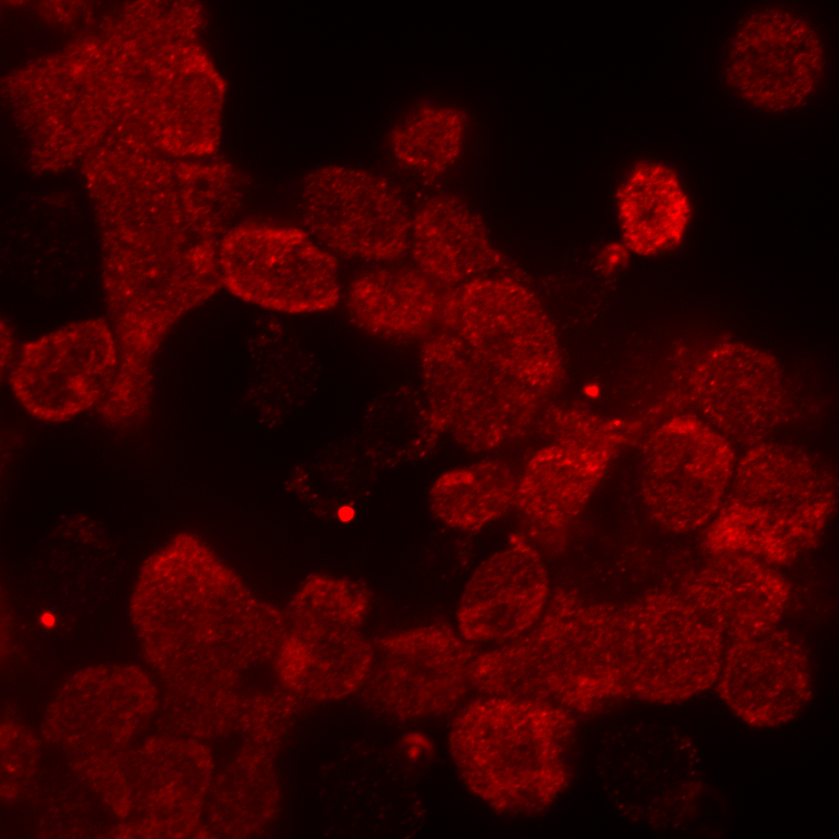

Supplement: Supplementary file 10 — Source data Fig. 4 [file 44318_2024_236_MOESM10_ESM.zip › Figure 4_final_submission_V3/Figure 4G/LPS+dA dT ASC.tif]

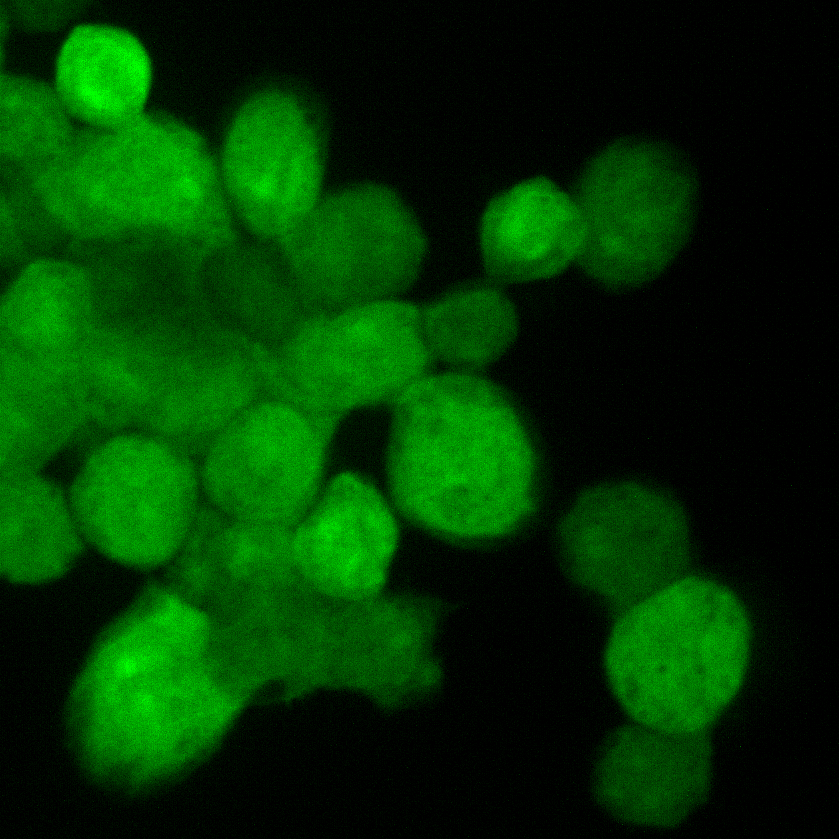

Supplement: Supplementary file 10 — Source data Fig. 4 [file 44318_2024_236_MOESM10_ESM.zip › Figure 4_final_submission_V3/Figure 4G/Mock GFP-ZNFX1.tif]

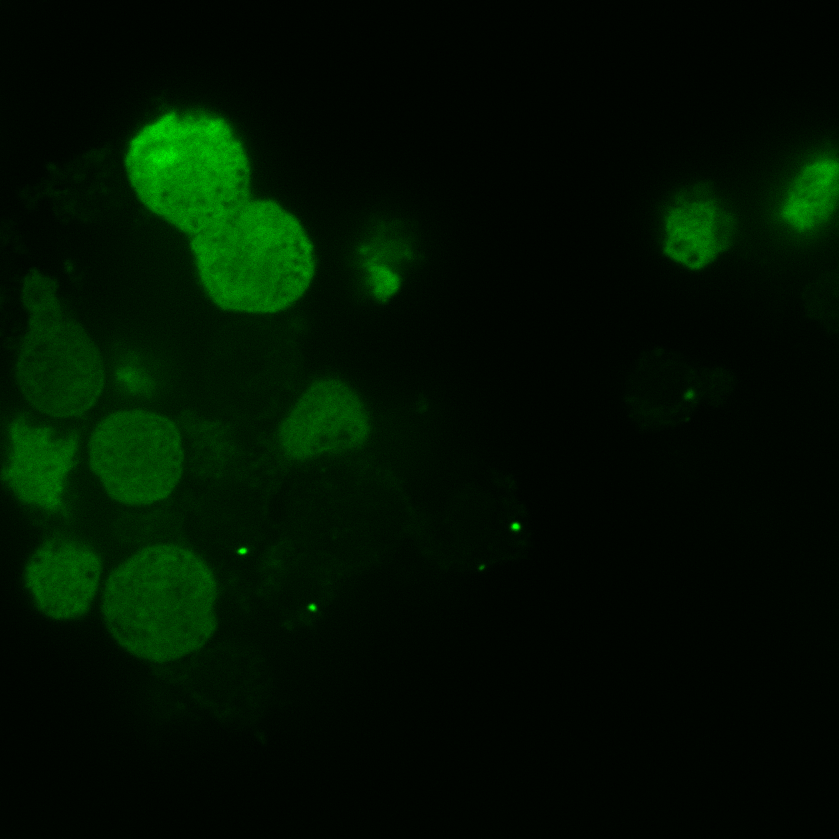

Supplement: Supplementary file 10 — Source data Fig. 4 [file 44318_2024_236_MOESM10_ESM.zip › Figure 4_final_submission_V3/Figure 4G/LPS+Nig GFP-ZNFX1.tif]

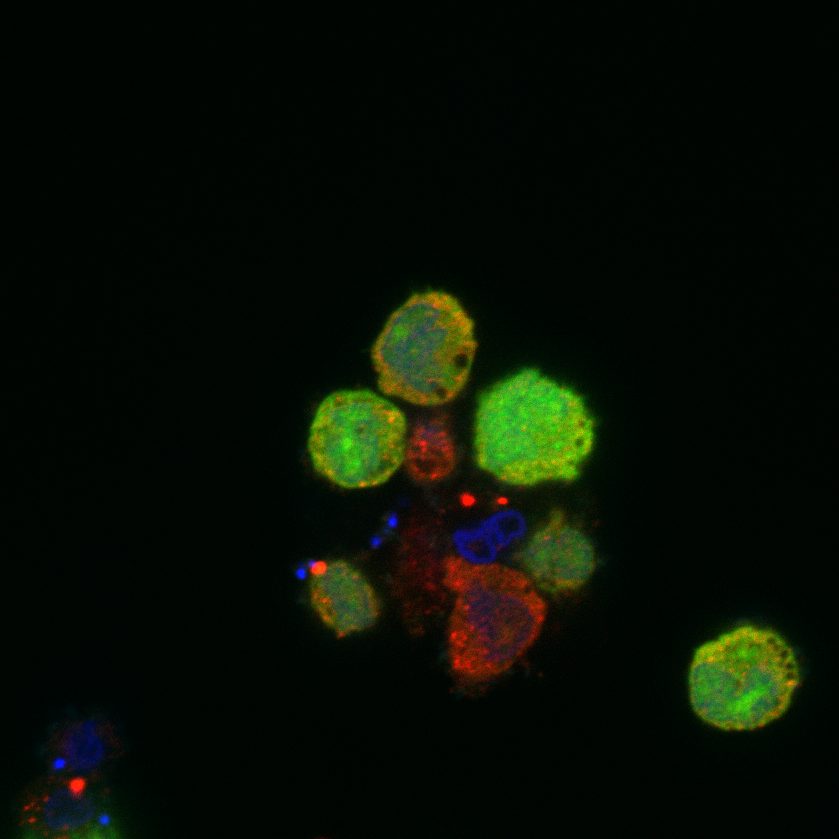

Supplement: Supplementary file 10 — Source data Fig. 4 [file 44318_2024_236_MOESM10_ESM.zip › Figure 4_final_submission_V3/Figure 4G/LPS+Salm Merge.tif]

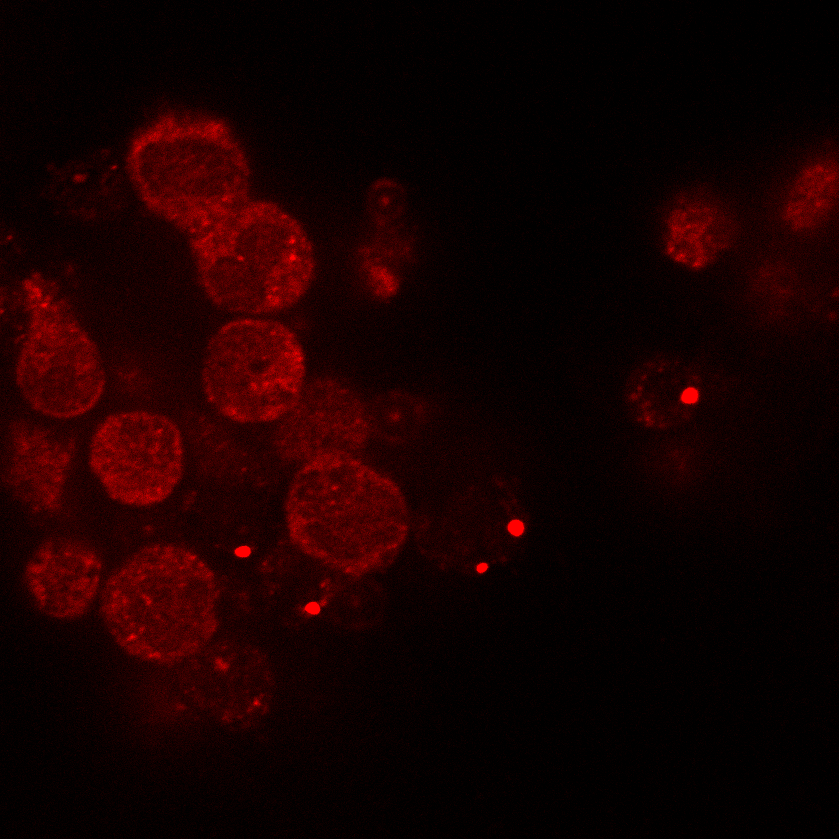

Supplement: Supplementary file 10 — Source data Fig. 4 [file 44318_2024_236_MOESM10_ESM.zip › Figure 4_final_submission_V3/Figure 4G/LPS+Nig ASC.tif]

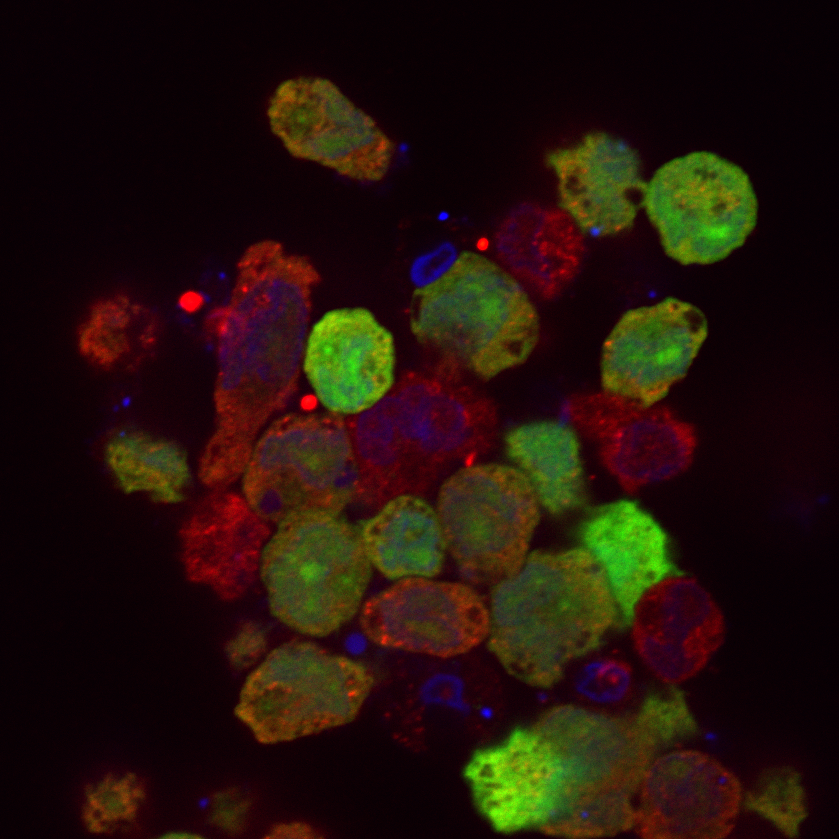

Supplement: Supplementary file 10 — Source data Fig. 4 [file 44318_2024_236_MOESM10_ESM.zip › Figure 4_final_submission_V3/Figure 4G/MDP Merge.tif]

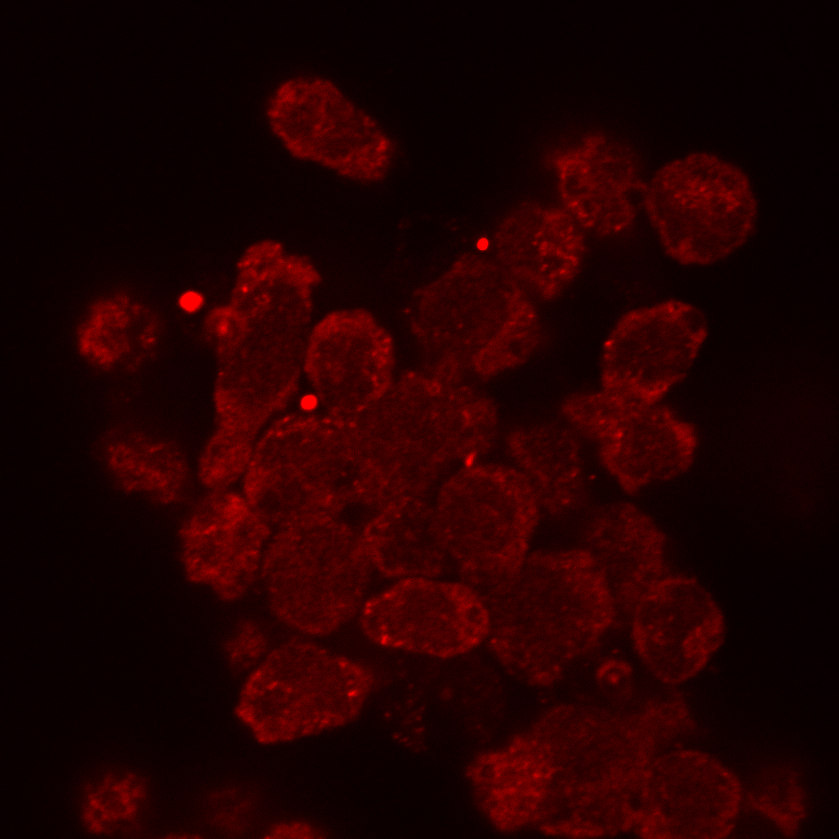

Supplement: Supplementary file 10 — Source data Fig. 4 [file 44318_2024_236_MOESM10_ESM.zip › Figure 4_final_submission_V3/Figure 4G/MDP ASC.tif]

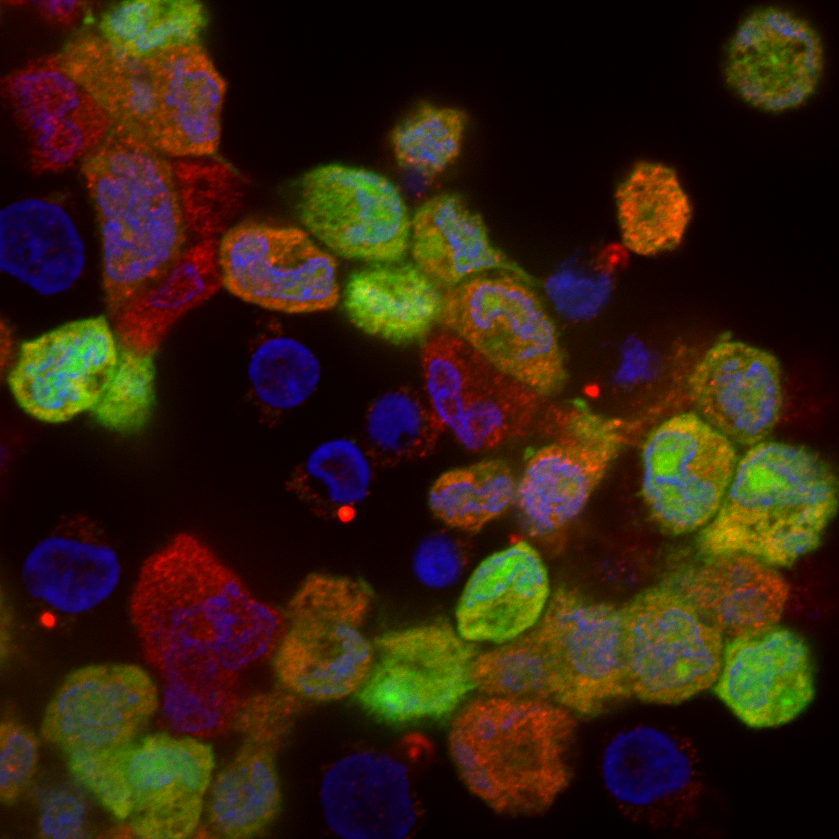

Supplement: Supplementary file 10 — Source data Fig. 4 [file 44318_2024_236_MOESM10_ESM.zip › Figure 4_final_submission_V3/Figure 4G/LPS+dA dT Merge.tif]

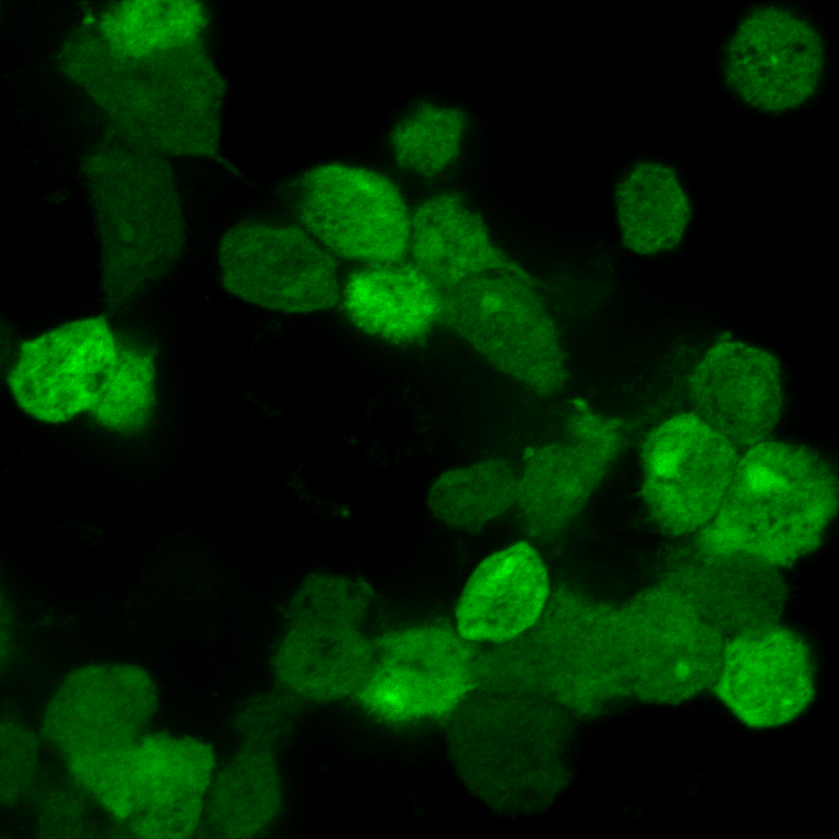

Supplement: Supplementary file 10 — Source data Fig. 4 [file 44318_2024_236_MOESM10_ESM.zip › Figure 4_final_submission_V3/Figure 4G/LPS+dA dT GFP-ZNFX1.tif]

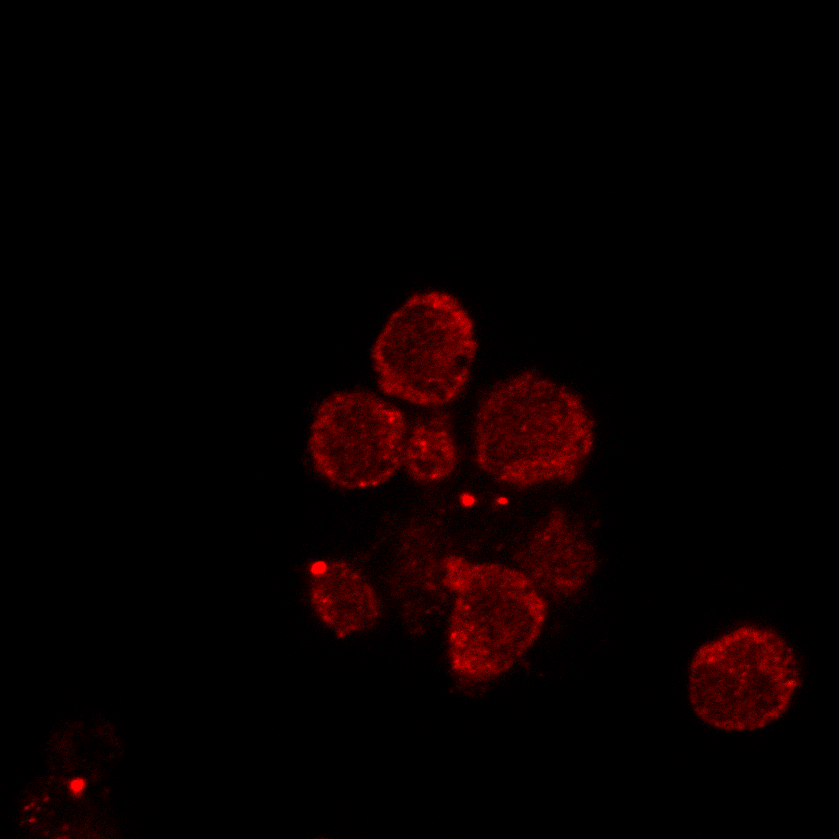

Supplement: Supplementary file 10 — Source data Fig. 4 [file 44318_2024_236_MOESM10_ESM.zip › Figure 4_final_submission_V3/Figure 4G/LPS+Salm ASC.tif]

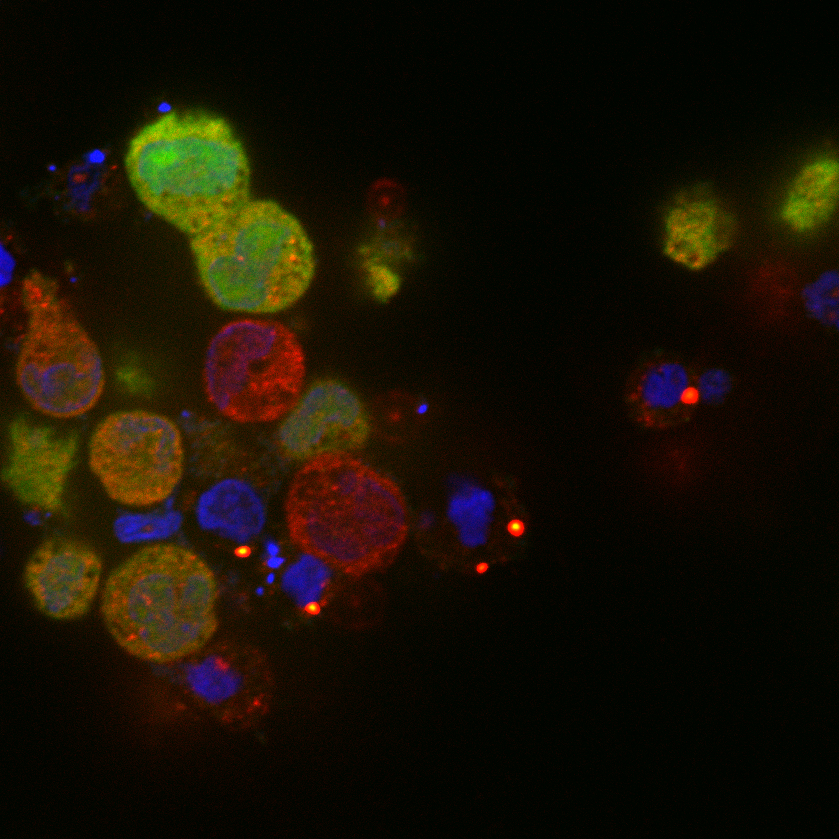

Supplement: Supplementary file 10 — Source data Fig. 4 [file 44318_2024_236_MOESM10_ESM.zip › Figure 4_final_submission_V3/Figure 4G/LPS+Nig Merge.tif]

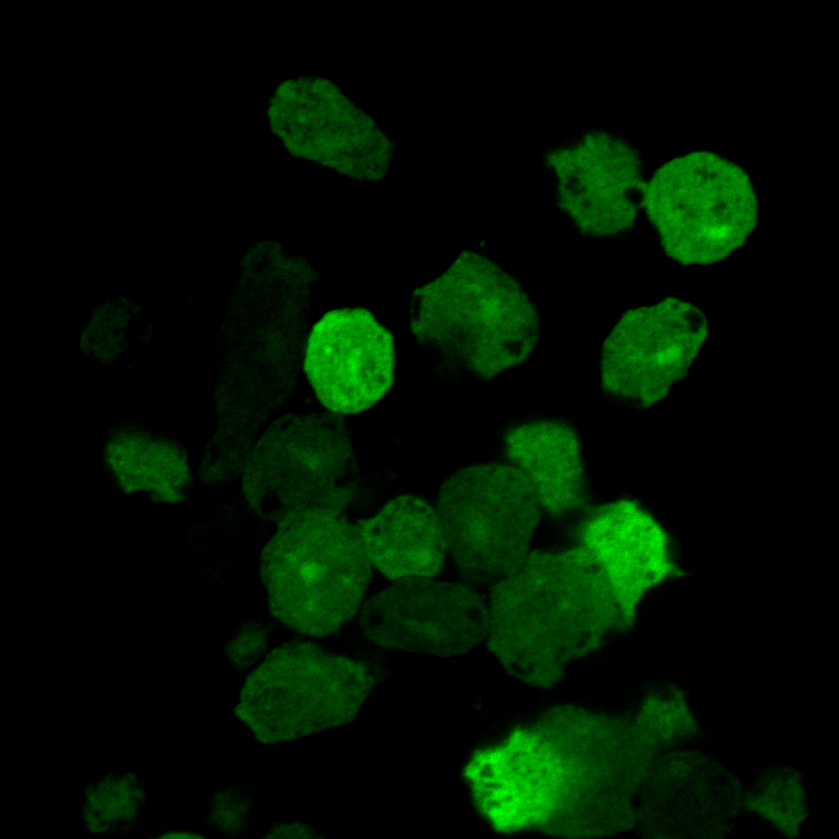

Supplement: Supplementary file 10 — Source data Fig. 4 [file 44318_2024_236_MOESM10_ESM.zip › Figure 4_final_submission_V3/Figure 4G/MDP GFP-ZNFX1.tif]

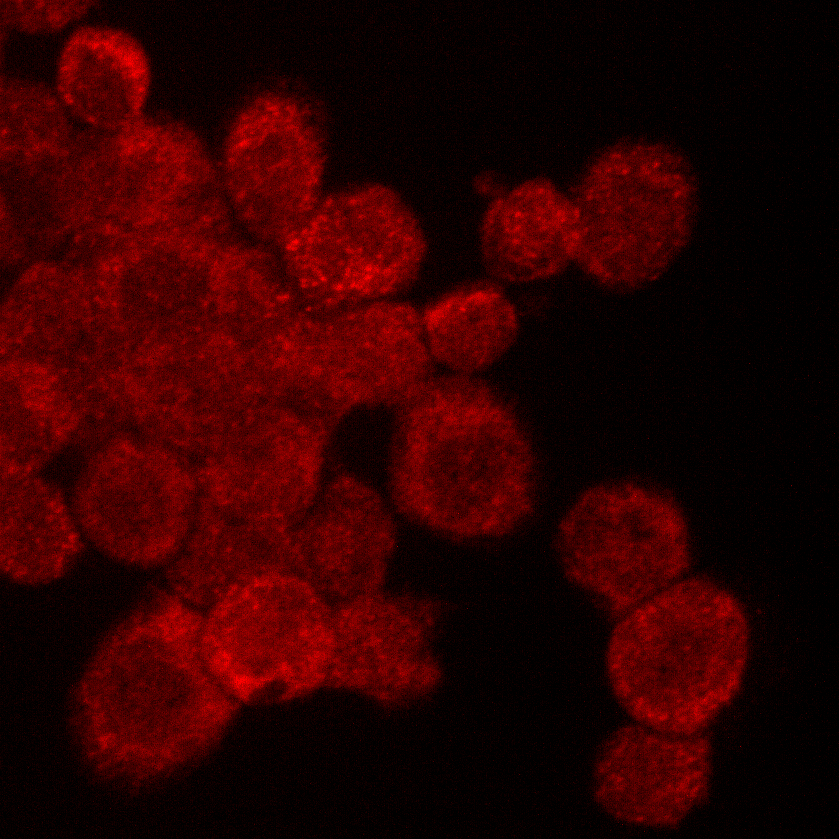

Supplement: Supplementary file 10 — Source data Fig. 4 [file 44318_2024_236_MOESM10_ESM.zip › Figure 4_final_submission_V3/Figure 4G/Mock ASC.tif]

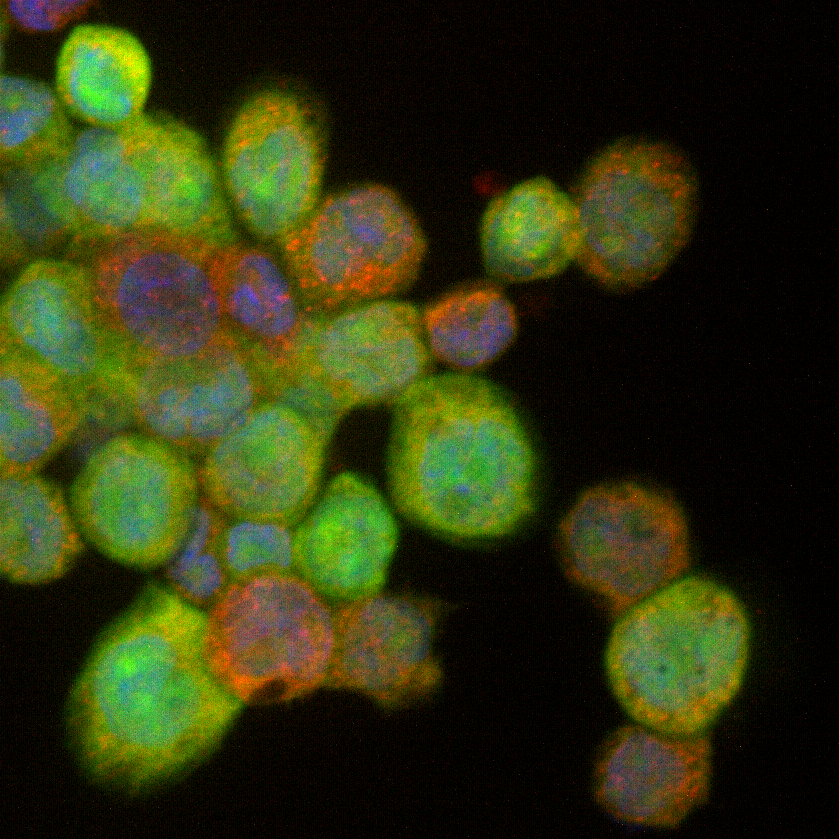

Supplement: Supplementary file 10 — Source data Fig. 4 [file 44318_2024_236_MOESM10_ESM.zip › Figure 4_final_submission_V3/Figure 4G/Mock Merge.tif]
