# Supplementary material for: The human disease-associated gene ZNFX1 controls inflammation through inhibition of the NLRP3 inflammasome
Source: EMBO J. 2024 Sep 27;43(22):9. doi: 10.1038/s44318-024-00236-9 (PMC11574294; doi:10.1038/s44318-024-00236-9)
Supplement: Supplementary file 15 — Expanded View Figures [file 44318_2024_236_MOESM15_ESM.pdf]

Expanded View Figures

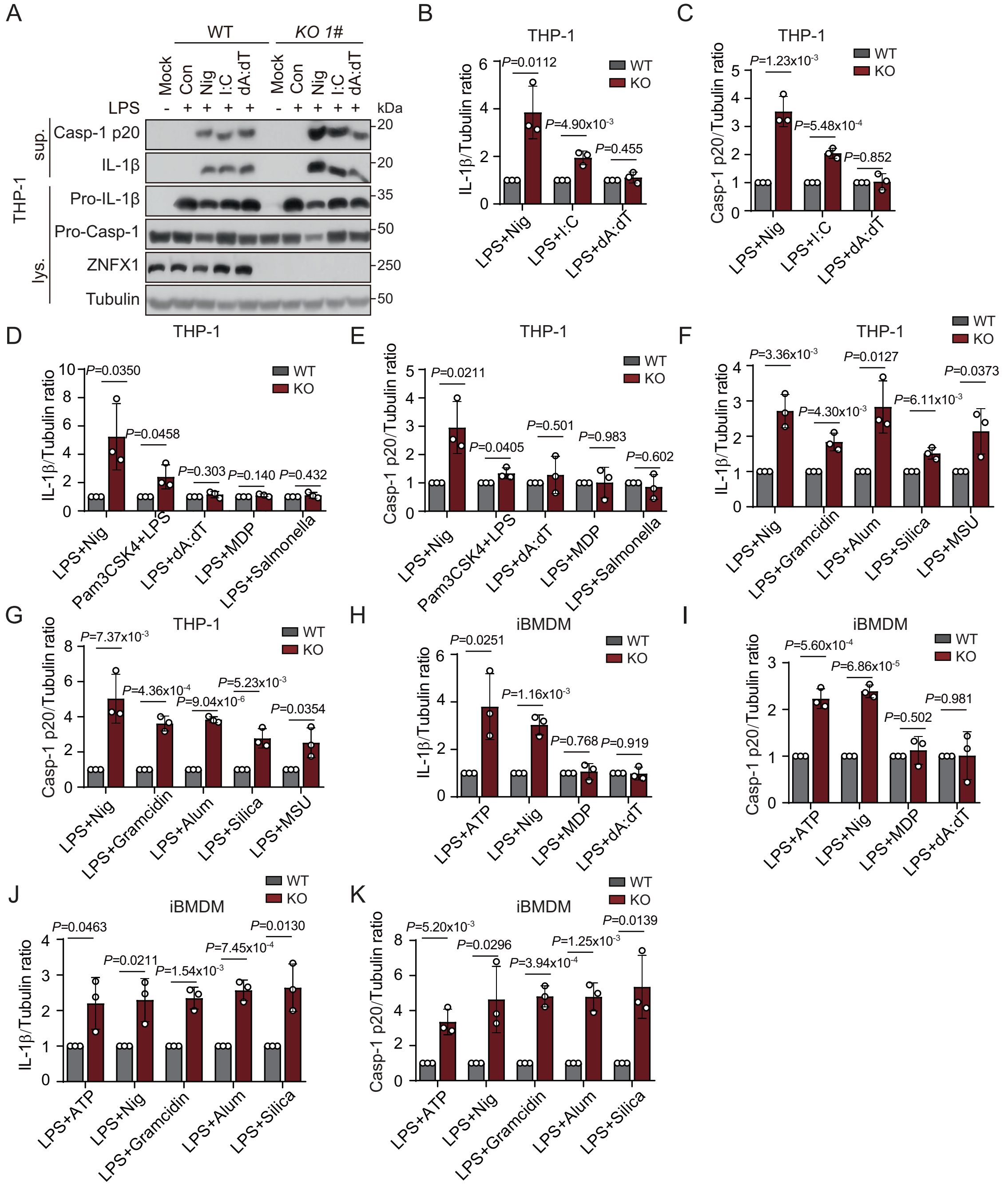

Figure EV1. ZNFX1 specifically suppresses the activation of the NLRP3 inflammasome in vitro (related to Fig. 1).

(**A**) LPS-primed wildtype (WT) or *ZNFX1* knockout (KO) THP-1-derived macrophages were treated with the indicated inflammation agonists, caspase-1 and IL-1β in the supernatant (sup.) and cell lysate (Lys.) were separated by SDS-PAGE and immunoblotted with the indicated antibodies. Mock represents macrophages primed with PBS without further stimulation. Con control, Nig nigericin, I:C poly(I:C), dA:dT, poly(dA:dT). (**B**, **C**) Quantification of IL-1β and caspase-1 P20 protein levels in (**A**). $n = 3$ biological replicates, error bar, ±s.d. (**D–G**) Quantification of IL-1β and caspase-1 P20 protein levels in Fig. 1A, B. $n = 3$ biological replicates, error bar ± s.d. (**H–K**) Quantification of IL-1β and caspase-1 P20 protein levels in Fig. 1C, D. $n = 3$ biological replicates, error bar, ±s.d. Student's *t*-test, two-tailed for (**B–K**). For each biological replicate, band intensity was measured using ImageJ. The WT control was set to 1, and the ratio for *ZNFX1* KO cells was calculated by dividing their intensity by the corresponding WT control intensity. Source data are available online for this figure.

 © The Author(s)

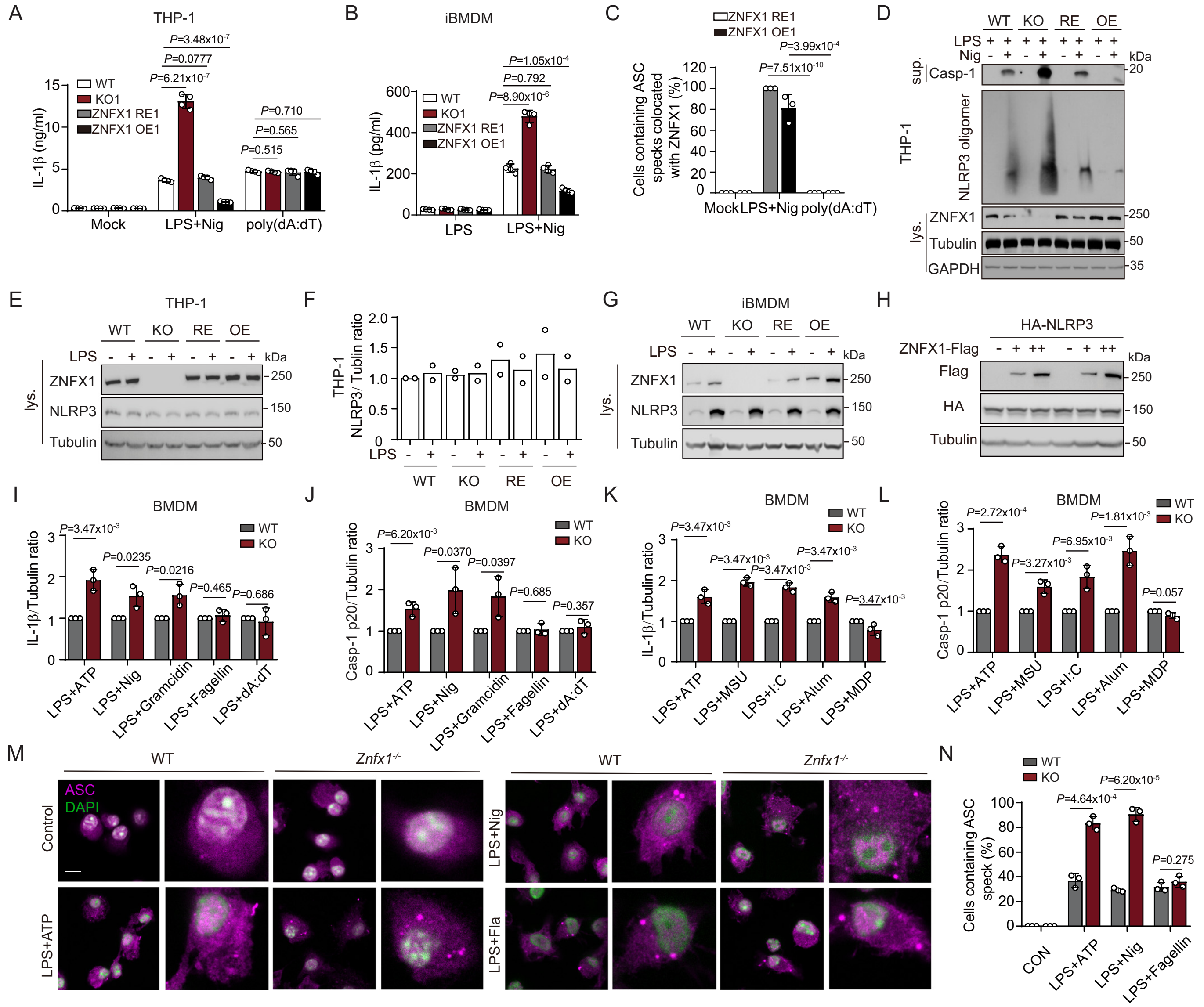

Figure EV2. ZNFX1 inhibits the assembly of mature NLRP3 inflammasome.

(**A**, **B**) ELISA detection of IL-1β in the supernatant as shown in Fig. 1G, H. Student's *t*-test, two-tailed. **C** Quantification of the percentage of cells with ASC specks containing GFP-ZNFX1 as shown in Fig. 1E. Student's *t*-test, two-tailed. (**D**) SDD-AGE analysis of NLRP3 oligomerization in the lysates of nigericin-treated macrophages. (**E**, **F**) Immunoblotting to detect indicated proteins in WT, *ZNFX1* KO, *ZNFX1* rescue (RE), and *ZNFX1* overexpression (OE) THP-1 derived macrophage with or without LPS priming (**E**). Quantification of NLRP3 protein level (**F**). $n = 2$ biological replicates. For each biological replicate, band intensity was measured using ImageJ. The WT control was set to 1, and the ratio for *ZNFX1* KO cells was calculated by dividing their intensity by the corresponding WT control intensity. (**G**) Immunoblotting to detect indicated proteins in WT, *ZNFX1* KO, *ZNFX1* RE, and *ZNFX1* OE IBMDM cells with or without LPS priming. (**H**) HeLa cells were transfected with HA-NLRP3 and increased level 3xFLAG-ZNFX1. Immunoblotting was used to detect indicated proteins. (**I–L**) Quantification of IL-1β and caspase-1 P20 protein level as shown in Fig. 1I, J. $n = 3$ biological replicates. Error bars represent ± s.d. Student's *t*-test, two-tailed. For each biological replicate, band intensity was measured using ImageJ. The WT control was set to 1, and the ratio for *ZNFX1* KO cells was calculated by dividing their intensity by the corresponding WT control intensity. (**M**) Primed WT and *Znfx1* BMDM cells were treated with the indicated inflammasome agonist. ASC speck was detected with anti-ASC antibody followed by Alex fluor-568 conjugated secondary antibody. Scale bar, 10 µm. (**N**) Quantification of the percentage of cells containing ASC specks in (**M**). $n = 3$ biological replicates. Error bars represent ± s.d. For each replicate, 100 cells were quantified. Student's *t*-test, two-tailed. Source data are available online for this figure.

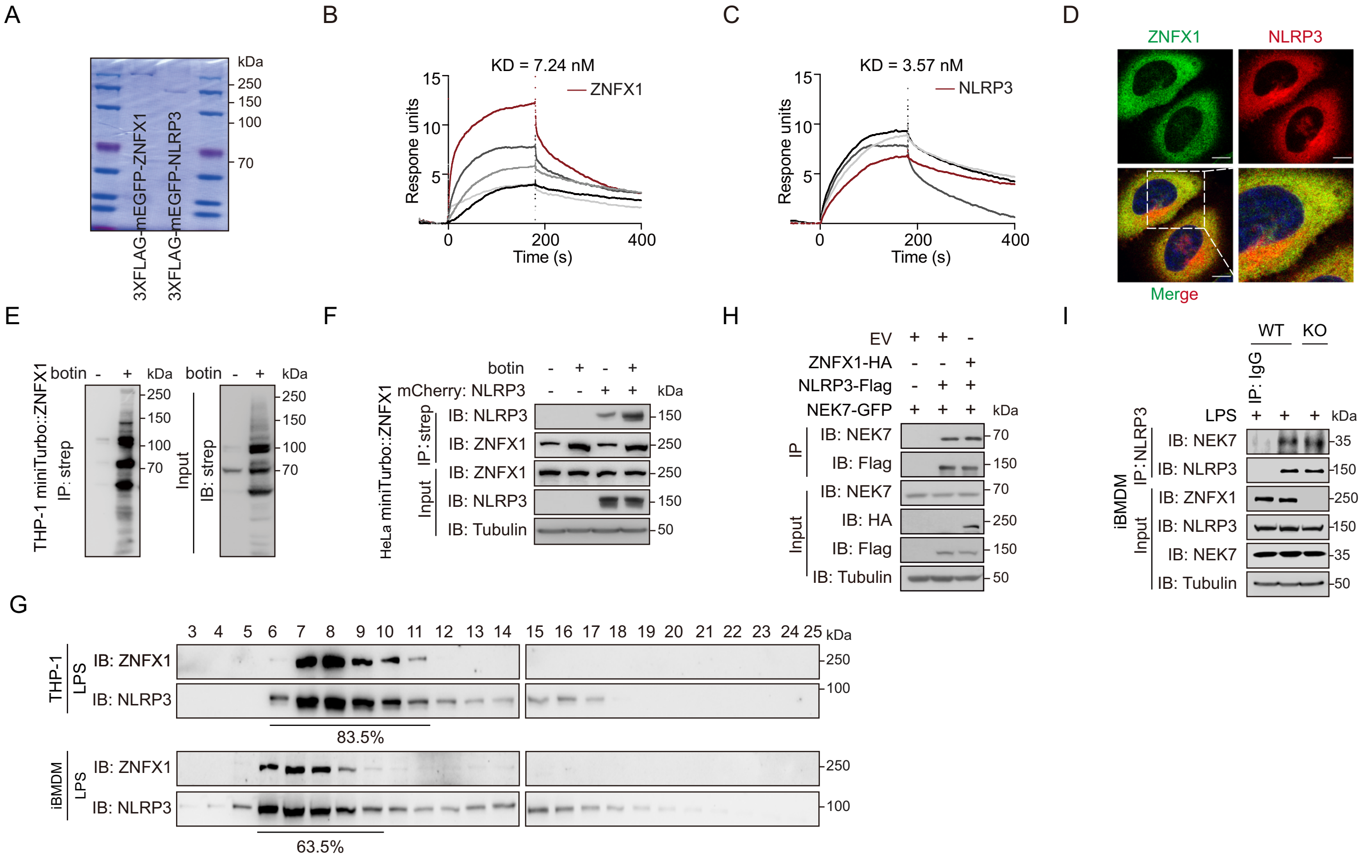

Figure EV3. ZNFX1 interacts with NLRP3 related to Fig. 3.

(**A**) Coomassie brilliant blue staining of purified 3xFLAG-GFP-ZNFX1 and 3xFLAG-GFP-NLRP3 proteins. (**B, C**) Purified 3xFLAG-ZNFX1 or 3xFLAG-NLRP3 proteins were immobilized, and proteins associated with the immobilized proteins were analyzed using surface plasmon resonance (SPR). (**D**) Fluorescence imaging of HeLa cells stably expressed GFP-ZNFX1 and mCherry-NLRP3. Scale bar, 10 μm. (**E**) Immunoblot analysis of proteins in cell lysate and miniTurbo-ZNFX1 catalyzed biotinylated species from THP-1 derived macrophages before and after adding biotin substrate using strep-HRP. (**F**) HeLa cells stably expressing miniTurbo-ZNFX1 or miniTurbo-ZNFX1 and mCherry-NLRP3 were treated with biotin to initiate proximity biotin labeling. Proteins from cell lysate and those enriched by streptavidin were resolved by SDS-PAGE and detected by immunoblot. (**G**) Proteins from resting THP-1-derived macrophages or iBMDM were separated using a size-exclusion Column. Eluted protein fractions were detected by immunoblot. (**H**) NEK7-GFP was co-expressed with empty vector (EV), HA-ZNFX1, or FLAG-NLRP3 in HEK293T cells. Coimmunoprecipitation analysis of indicated proteins was performed using α-GFP agarose beads. (**I**) Cell lysate from WT or *Znfx1* KO iBMDM cells was precipitated with NLRP3. Immunoblot analysis of proteins in a cell lysate or co-precipitated fraction using indicated antibodies. Source data are available online for this figure.

 © The Author(s)

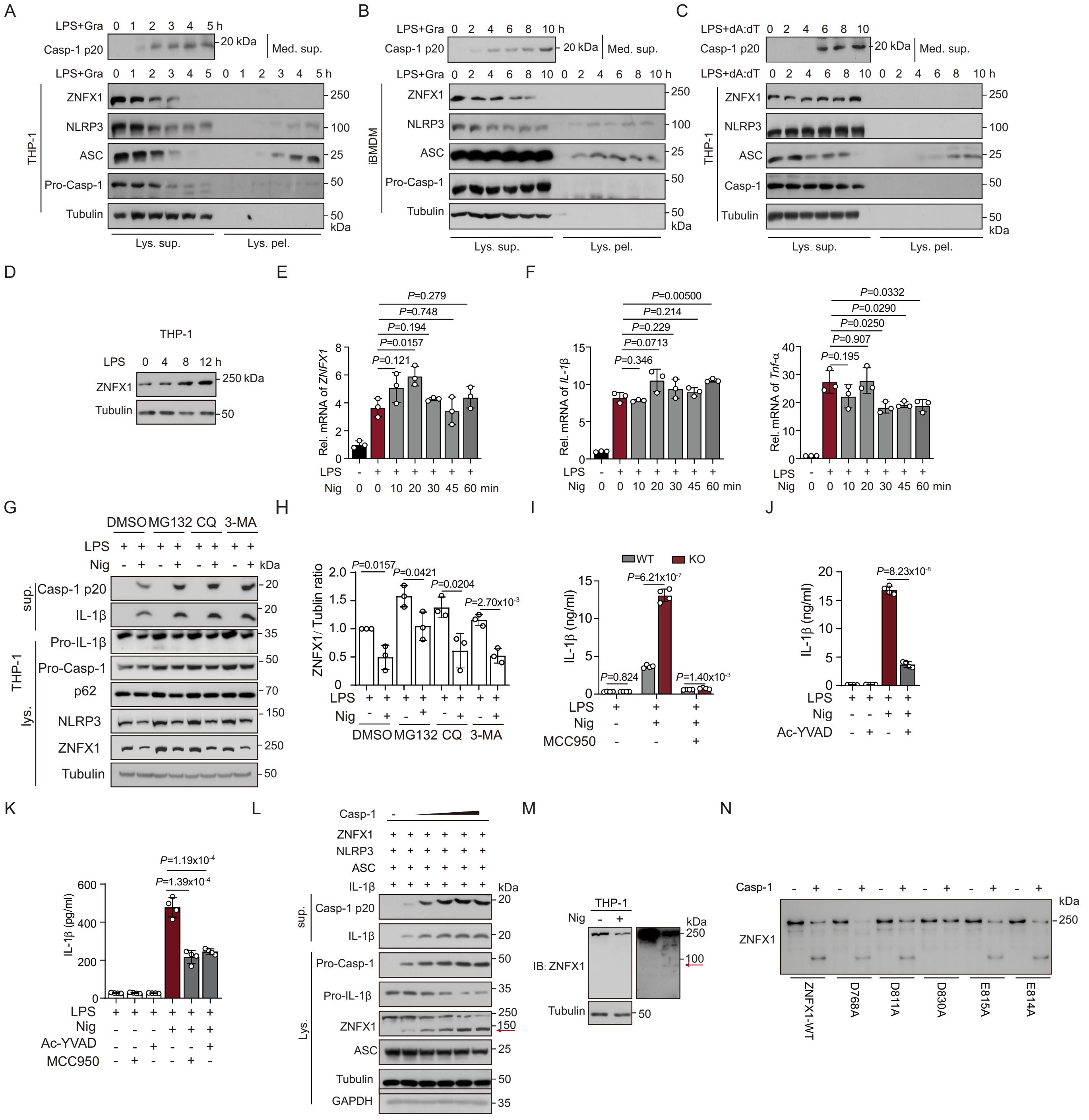
A
B
C
D
E
F
G
H
I
J
K
L
M
N
LPS+Gra
Casp-1 p20
Med. sup.
Lys. sup.
Lys. pel.
ZNFX1
NLRP3
ASC
Pro-Casp-1
Tubulin
THP-1
iBMDM
LPS+dA:dT
Casp-1
Rel. mRNA of ZNFX1
Rel. mRNA of IL-1β
Rel. mRNA of Tnf-α
LPS
Nig
DMSO MG132 CQ 3-MA
Pro-IL-1β
p62
IL-1β
ZNFX1/ Tublin ratio
IL-1β (ng/ml)
IL-1β (pg/ml)
WT
KO
MCC950
Ac-YVAD
GAPDH
IB: ZNFX1
ZNFX1-WT
D768A
D811A
D830A
E815A
E814A

Figure EV4. The decreased *ZNFX1* protein level in response to NLRP3 inflammasome activation is largely posttranscriptional and independent of the ubiquitin-proteasome and autophagy pathway.

(**A**, **B**) LPS-primed THP-1-derived macrophages (**A**) or iBMDM (**B**) were treated with gramicidin for the indicated time. Proteins from medium supernatant, cell lysate supernatant, and pellet were detected by immunoblot. (**C**) LPS-primed THP-1 derived macrophages were transfected with poly (dA:dT). Proteins from medium supernatant, cell lysate supernatant, and pellet post-transfection were detected by immunoblot. (**D–F**) THP-1 derived macrophages were primed with LPS and treated with 10 μM nigericin for the indicated time, the protein level of *ZNFX1* (**D**), mRNA level of *ZNFX1* (**E**), and mRNA level of *IL-1β* (**F**) and *TNF-α* (**F**) were measured by immunoblotting or quantitative reverse transcription PCR (qRT-PCR). $n = 3$ biological replicates, mean ± s.d., Student's *t*-test, two-tailed. (**G**, **H**) LPS-primed THP-1 derived macrophages were pretreated with proteasome inhibitor MG132 (10 μM), autophagy inhibitor 3-MA (20 mM), or CQ (100 μM) for 4 h, and then stimulated with nigericin. Proteins from medium supernatant and cell lysate were detected by immunoblot (**G**). Quantification of *ZNFX1* protein was performed using ImageJ (**H**). $n = 3$ biological replicates. Error bars represent ± s.d. Student's *t*-test, two-tailed. For each biological replicate, band intensity was measured using ImageJ. The WT control was set to 1, and the ratio for *ZNFX1* KO cells was calculated by dividing their intensity by the corresponding WT control intensity. (**I–K**) Primed WT THP-1 derived macrophages (**I–J**) or iBMDM (**K**) were subjected to LPS or nigericin treatment with or without MCC950 or caspase-1 inhibitor (Ac-YVAD-cmk) pretreatment. Proteins from culture medium supernatant were detected by ELISA. $n = 3$ biological replicates, mean ± s.d., Student's *t*-test, two-tailed. (**L**) Indicated proteins were co-expressed in HeLa cells. Proteins from medium supernatant and cell lysate were detected by immunoblot. (**M**) Proteins from cell lysate of LPS + nigericin-treated THP-1 cells were detected by immunoblot. The red arrow indicates putative cleaved *ZNFX1* fragments. (**N**) Purified 3xFLAG-mEGFP-ZNFX1 with or without potential caspase-1 cleavage sites mutated were incubated with caspase-1 protein. Products were detected by immunoblot using α-FLAG antibody. This is an independent biological replicate related to Fig. 6D. Source data are available online for this figure.

 © The Author(s)

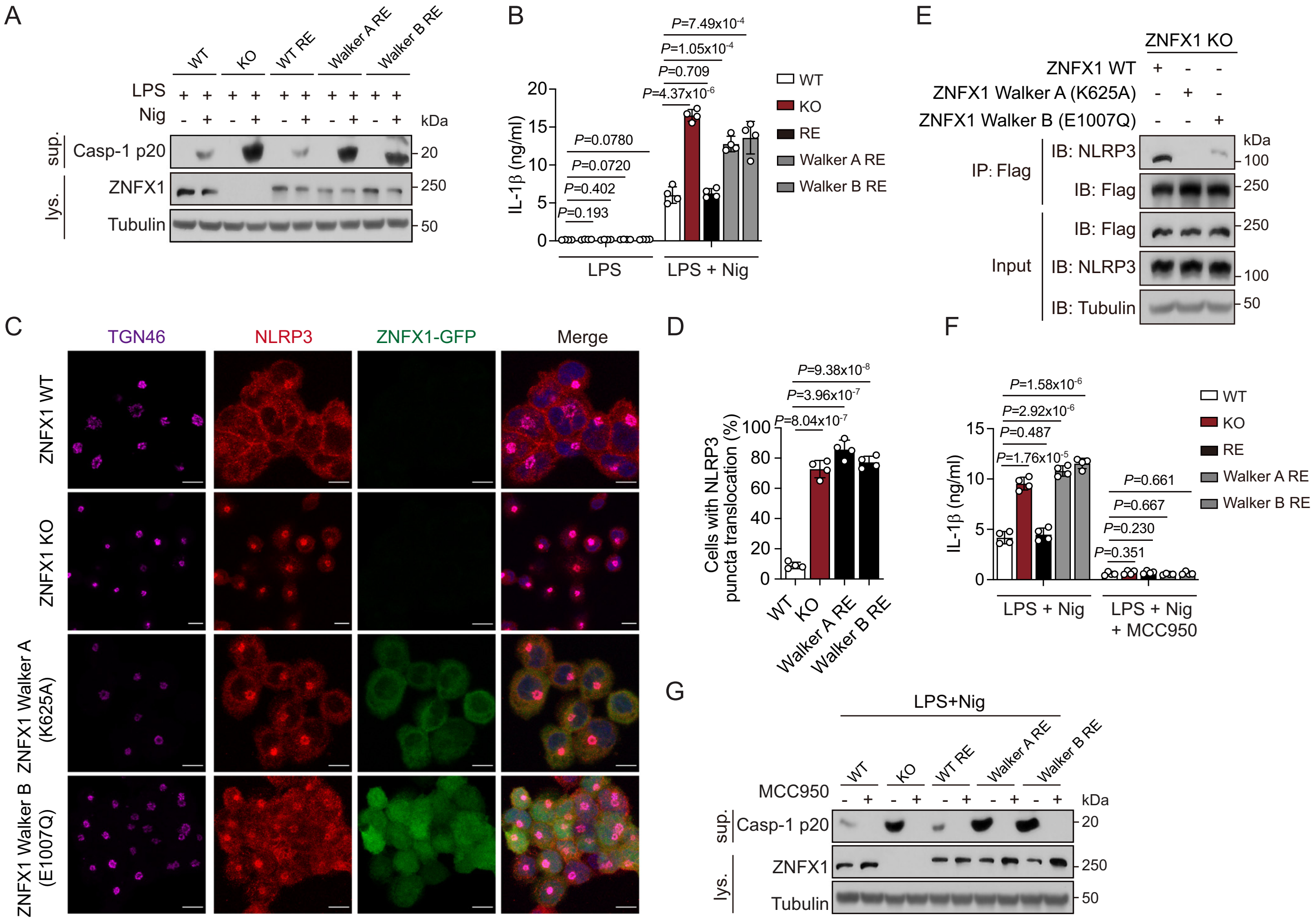

Figure EV5. The helicase activity of *ZNFX1* is critical for NLRP3 inflammasome inhibition.

(**A**, **B**) WT *ZNFX1* or *ZNFX1* containing Walker A or Walker B motif mutations were introduced back to *ZNFX1* KO THP-1 cells by lentivirus-mediated delivery. Cells were primed with LPS and stimulated with nigericin. Proteins from medium supernatant or cell lysate were examined with immunoblot (**A**) and ELISA (**B**). $n = 4$ biological replicates, mean ± s.d., Student's *t*-test, two-tailed. (**C**) WT, *ZNFX1* KO, or *ZNFX1* KO cells complemented with *ZNFX1* harboring helicase mutations were fixed and stained with anti-TGN46 and anti-NLRP3 antibodies, followed by Alex fluor-647 and Alex fluor-568 conjugated secondary antibodies, respectively. Scale bar, 10 μm. (**D**) Quantification of the percentage of cells with NLRP3's TGN translocation from 100 cells in (**C**). $n = 3$, mean ± s.d., two-sided Student's *t*-test. (**E**) Coimmunoprecipitation analysis of NLRP3 with WT or helicase mutants of ZNFX1. Proteins in input cell lysate, as well as precipitation, were detected with indicated antibodies. (**F**, **G**) Cells with indicated genotype were primed with LPS and treated with nigericin, with or without MCC950 pretreatment, NLRP3 inflammation activation was measured by ELISA in (**F**) and immunoblot in (**G**). $n = 4$ biological replicates, mean ± s.d., Student's *t*-test, two-tailed. Source data are available online for this figure.

© The Author(s)
